# Facilely Achieved Self‐Biased Black Silicon Heterojunction Photodiode with Broadband Quantum Efficiency Approaching 100%

**DOI:** 10.1002/advs.202203234

**Published:** 2022-10-17

**Authors:** Yibo Zhang, Joel Y. Y. Loh, Nazir P. Kherani

**Affiliations:** ^1^ The Edward S. Rogers Sr. Department of Electrical and Computer Engineering University of Toronto 10 King's College Road Toronto Ontario M5S 3G4 Canada; ^2^ Department of Materials Science and Engineering University of Toronto 184 College Street Toronto Ontario M5S 3E4 Canada

**Keywords:** heterojunction photodiode, nanostructured black silicon, quantum efficiency, self‐biased photodetection, space charge effect

## Abstract

Photodiodes are fundamental components in modern optoelectronics. Heterojunction photodiodes, simply configured by two different contact materials, have been a hot research topic for many years. Currently reported self‐biased heterojunction photodiodes routinely have external quantum efficiency (EQE) significantly below 100% due to optical and electrical losses. Herein, an approach that virtually overcomes this 100% EQE challenge via low‐aspect‐ratio nanostructures and drift‐dominated photocarrier transport in a heterojunction photodiode is proposed. Broadband near‐ideal EQE is achieved in nanocrystal indium tin oxide/black silicon (*nc*‐ITO/*b*‐Si) Schottky photodiodes. The *b*‐Si comprises nanostalagmites which balance the antireflection effect and surface morphology. The built‐in electric field is explored to match the optical generation profile, realizing enhanced photocarrier transport over a broadband of photogeneration. The devices exhibit unprecedented EQE among the reported leading‐edge heterojunction photodiodes: average EQE surpasses ≈98% for wavelengths of 570–925 nm, while overall EQE is greater than ≈95% from 500 to 960 nm. Further, only elementary fabrication techniques are explored to achieve these excellent device properties. A heart rate sensor driven by nanowatt faint light is demonstrated, indicating the enormous potential of this near‐ideal *b*‐Si photodiode for low power consuming applications.

## Graphical abstract

This work presents simply fabricated black silicon heterojunction photodiodes with near‐100% broadband quantum efficiency. Nanostalagmites are fabricated for an easy‐to‐passivate surface with high optical absorption. Nanocrystal indium tin oxide contact and implementation of a deep‐well depletion region enable effective photocarrier transport and extraction. The self‐biased device exhibits >98% average external quantum efficiency from 570 to 925 nm wavelength.

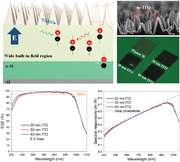

## 1 Introduction

Self‐biased semiconductor photodetectors, converting incident photons to electrical signals without an applied voltage bias, have been a topic of much research interest considering the continual trend in producing low power consumption optoelectronic devices and products. Within this framework, the low processing cost and the ease of integration have inspired the development of various self‐biased Si heterojunctions with high performance.^[^ ^1^, ^2^, ^3^, ^4^, ^5^, ^6^ ^]^ However, the simply constructed heterojunction silicon photodiodes invariably exhibit an external quantum efficiency (EQE) significantly below 100%.^[^ ^7^, ^8^, ^9^, ^10^ ^]^

The advent of black silicon (*b*‐Si) has led to the development of innovative photodiodes and solar cells that yield high quantum efficiency over a broadband of wavelengths, having elegant combinations of advanced surface passivation technologies, light trapping/antireflection structures, and device constructs.^[^ ^11^, ^12^, ^13^, ^14^, ^15^, ^16^, ^17^ ^]^ Savin et al., e.g., reported a PIN *b*‐Si photodiode with an average EQE exceeding ≈96% for wavelengths ranging from 250 to 950 nm, realized by reactive ion etching fabrication of nanoneedles, together with an induced junction via atomic layer deposition (ALD) of Al_2_O_3_ passivation dielectrics.^[^ ^13^ ^]^ Notably, the reported advanced PIN *b*‐Si photodiodes exhibit above‐100% EQE, in fact up to >130% EQE at short wavelengths,^[^ ^12^, ^13^ ^]^ breaking the barrier that one photon can only generate one electron–hole pair. These state‐of‐the‐art Si PIN devices, however, require several relatively complicated fabrication techniques such as ALD, plasma etching, photolithography, and/or high‐temperature dopant diffusion processes for high thermal budgets. Meanwhile, various alternative strategies have drawn recent research focus toward the development of a single contact material that can be simply integrated within nanostructured Si morphology to achieve high quantum efficiency heterojunctions.^[^ ^4^, ^18^, ^19^, ^20^, ^21^ ^]^ For example, Liang et al., reported a self‐biased poly(3,4‐ethylenedioxythiophene):poly(styrenesulfonate) (PEDOT:PSS)/Si nanowire photodetector with 60–80% EQE from 400 to 900 nm.^[^ ^21^ ^]^ Notwithstanding these reported advances, present‐day heterojunction photodetectors continue to fall short of achieving unity EQE across the visible and near‐infrared (NIR) wavelengths due to nonideal absorption, nonoptimal charge carrier transport, photocarrier recombination losses in bulk and at interfaces, severe parasitic optical absorption in various contact layers and more. The near‐100% broadband EQE in a self‐biased heterojunction photodiode has not been achieved yet. As such, the principal motivation of the present work is to design and fabricate a facile heterojunction photodiode with just a single contact material yet realizing high device performance comparable to that of state‐of‐the‐art *b*‐Si PIN photodiodes.

Herein, we report a facile self‐biased nanocrystal indium tin oxide/black silicon (*nc*‐ITO/*b*‐Si) Schottky heterojunction photodiode with near‐ideal performance through the integration of innovative device design, interfacial heterojunction material processing, and nanostructuring of the silicon surface. A facile metal‐assisted chemical etching (MACE) process is utilized to fabricate nanostalagmite structures on Si. Broadband high optical absorption is addressed principally via these nanostalagmites that deliver a delicate balance between low optical reflectance and a surface morphology with a low ratio of actual to projected areas. A Schottky heterojunction contact is adopted to yield a built‐in electric field that extends into the silicon absorber well beyond tens of micrometers so as to match the optical generation profile in the absorber, thereby enabling effective charge carrier transport for both short and long wavelength photogeneration. A high‐quality interface is achieved via a combination of the contact‐induced surface electric field and the native Si oxide chemical passivation, which together effectively preclude the need for elaborate fabrication processes.^[^ ^22^ ^]^ Only basic technologies are utilized to produce the single contact *b*‐Si photodiode. From 570 to 925 nm wavelengths, the self‐biased heterojunction photodiode exhibits average broadband EQE of greater than ≈98%; and, from 500 to 960 nm, the overall EQE is over ≈95%. As an application example, we demonstrate the *b*‐Si photodiode as a sensitive heart rate sensor which functions across a broadband of visible and NIR wavelengths of 470–1050 nm. Further, the sensor is sensitive to a mere nanowatt level illumination power, making it possible for heart rate sensors to operate with low power‐consuming light sources. The near‐unity EQE, self‐biased, facilely fabricated *b*‐Si heterojunction photodiode opens a path to its widespread application in a range of miniature light‐weight self‐biased mechanical, optical, electronic devices and products.

## 2 Results and Discussion

### 2.1 Device Structures and Topological Analysis

**Figure 1:**
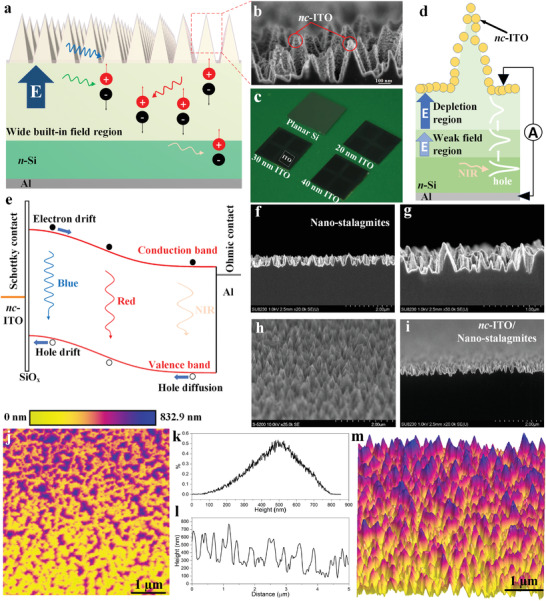
a) 3D device structure. b) High‐magnification cross‐sectional HRSEM image of *nc*‐ITO/nanostalagmites. c) Optical photograph of *b*‐Si devices with different ITO film thicknesses in comparison with untreated planar Si surface. d) 2D illustration of the device. e) Energy band diagram of the device. f) Low‐magnification cross‐sectional HRSEM image of bare nanostalagmites on c‐Si. g) High‐magnification cross‐sectional HRSEM image of bare nanostalagmites. h) Tilted cross‐sectional HRSEM image of nanostalagmites on c‐Si without ITO. i) Low‐magnification cross‐sectional HRSEM image of *nc*‐ITO/nanostalagmites. j) 2D AFM image of nanostalagmites. k) Height distribution statistics. l) A typical depth profile across the scanning region. m) 3D AFM image illustrating surface morphology. (j) and (m) have the same scale bar.

The device structure is shown in **Figure** **1**a. Nanostalagmites are fabricated on high resistivity *n*‐Si (≈3000 ohm‐cm, ≈10^12^ cm^−3^ doping level) with ITO nanocrystals being the sputter‐deposited rectifying contact. Aluminum is sputter‐deposited on the backside of the device as the ohmic contact. The ITO layer serves as a Schottky contact and, considering its relatively high electrical conductivity, it also serves as a top transparent electrode. The deposition of a metal contact on top of ITO was avoided given the potential of introducing additional defect states at the interface, as well as maintaining fabrication simplicity. The *nc*‐ITO‐coated nanostalagmites are indicated by high‐resolution scanning electron microscope (HRSEM) in Figure 1b. A photograph of the actual *b*‐Si photodiodes with various thicknesses of ITO contacts compared with the un‐treated planar Si is shown in Figure 1c. As shown in Figure 1d, the built‐in electric field induced by the *nc*‐ITO contact extends from the surface of the nanostalagmites into the silicon absorber—this is the “strong‐field depletion region” (≈40 µm width, as shown in the simulation below), while the electric field region that extends to a depth of approximately a hundred micrometers is designated as the “weak field region.” More precisely, the device energy band diagram is explained in Figure 1e. A gently progressing but physically extensive band bending is realized within *b*‐Si, where drift‐dominated hole transport is indicated for photocarriers generated at both short (blue) and long (NIR) wavelengths. Under illumination, the photocarrier generation profile is related to the semiconductor refractive index and the antireflective nanostructures. For the device in this work, the Schottky contact and the correspondingly induced wide space charge region make it possible that most of the photocarriers, generated due to 400–960 nm wavelengths of the incident light, appear within the strong field region. For example, the absorption depth for Si at 960 nm wavelength is ≈74.6 µm, indicating that most of the photocarriers generated below 960 nm wavelength light are generated at tens of µm distance from the *b*‐Si surface. The wide depletion region (space charge region) and the electric field tailing region (weak field region) in this device serve to rapidly sweep and effectively extract the charge carriers. Additionally, the strong near‐surface electric field strength induced by the Schottky contact, and the proper interfacial chemical passivation effect associated with the native‐oxide‐based air‐annealing process,^[^ ^22^ ^]^ remarkably mitigate photocarrier loss to surface recombination. Cross‐sectional SEM images for bare nanostalagmite structures are shown in Figure 1f (low magnification) and Figure 1g (high magnification). In comparison with the rough surface prior to the removal of Au particles (Figure S1, in the Supporting Information), a noticeably smoother Si surface is obtained due to the surface polishing effect of radio corporation of America (RCA) cleaning.^[^ ^23^ ^]^ Tilt‐view cross‐sectional SEM image of the nanostalagmites (Figure 1h) illustrates a dense array of the nanostructures. A large area cross‐sectional SEM image of *nc*‐ITO (≈20 nm thickness, recorded by the thickness monitor of the sputtering system) coated *b*‐Si is shown in Figure 1i. See Figure 1b,i, the ITO thin film manifests itself as contiguous quasi‐uniform crystal islands distributed on the surface of the nanostalagmites. The surface morphology and height distribution of the nanostalagmites are investigated by atomic force microscopy (AFM). A 2D image of the height distribution is given in Figure 1j, where the nanostalagmites are quasi‐uniformly distributed with the height up to ≈833 nm. The statistical distribution of the height (Figure 1k) shows that most of the nanostalagmites lie in ≈400 to ≈600 nm height, a critical length scale for these nanostructures to provide a balance between antireflective effects and regular geometric distribution. The moderate height distribution and low geometrical aspect ratio of the nanostalagmites mitigate the need for complicated thin‐film deposition with absolute conformality, and as such provide a basis for facilely fabricated high‐performance photodiodes. A typical height profile of the nanostalagmites across 5 µm scanning length (Figure 1l) shows the distribution of height spanning ≈100 to ≈700 nm with a predominance of peaks within ≈300 to ≈600 nm range, consistent with the results in Figure 1k. The tapered profile of the stalagmites, evident in the 3D image in Figure 1m, provides optical impedance matching, thus contributing to absorption enhancement over short wavelengths. The “optical impedance matching” is explained in detail in the Supporting Information. The oblique sidewall geometry of the nanostalagmites and the resulting graded change in the optical refractive index enable high absorption while having a relatively small increase in the effective surface area. Therefore, this “easy‐to‐passivate” nanostructured surface allows for both high absorption and elimination of elaborate conformal passivation techniques.

**Figure 2:**
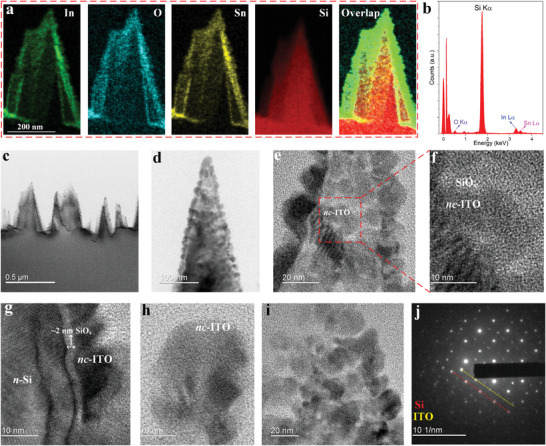
a) EDS elemental mapping of a sole *nc*‐ITO/nanostalagmite, where the elements indium (green), oxygen (cyan), tin (yellow), and silicon (red) are shown individually, and then all the elements are shown together. b) Elemental energy spectrum showing O K*α*, Si K*α*, In L*α*, and Sn L*α* peaks. c) HRTEM image of an array of *nc*‐ITO/nanostalagmites. d) HRTEM image of a sole *nc*‐ITO/nanostalagmite. e) High‐magnification image of the middle region of (d). f) High‐magnification image of a single *nc*‐ITO attached to a nanostalagmite, where the labeled white region is SiO*_x_*. g) HRTEM image of the sidewall region. ≈2 nm SiO*_x_* is observed. h) HRTEM image of a single particle of ITO, where highly ordered ITO lattice planes are observed. i) HRTEM image illustrating a close‐up view of *nc*‐ITO particles quasi‐uniformly distributed over the nanostalagmite surface. j) Diffraction pattern where the red dotted line corresponds to the Si lattice while the yellow dotted line to the ITO lattice.

Energy‐dispersive X‐ray spectroscopy (EDS) mapping and high resolution transmission electron microscopy (HRTEM) imaging of *nc*‐ITO/nano‐stalagmite structures are presented in **Figure** **2**. The elemental EDS mapping is shown in Figure 2a, where the elements indium (color green), oxygen (color cyan), and tin (color yellow) observably map out uniformly around the Si (color red) nanostalagmite. The elemental energy spectrum is plotted in Figure 2b. Si K*α* line dominates with a clear display of oxygen K*α*, indium L*α*, and tin L*α* lines peaking at ≈0.52, ≈3.29, and ≈3.50 keV, respectively. An HRTEM image of an array of ITO‐coated nanostalagmites is shown in Figure 2c. The stalagmite heights average ≈500 nm while the widths range from ≈200 to ≈300 nm, consistent with the AFM results in Figure 1k,l. HRTEM images for single *nc*‐ITO/nanostalagmite are shown in Figure 2d–j. Observation of the low (Figure 2d) and high (Figure 2e) magnification images reveals that the *nc*‐ITO film encapsulates the nanostalagmite in the form of contiguous crystals, consistent with the HRSEM results in Figure 1b. We note that upon annealing in the air, the ITO film undergoes lattice relaxation; further, minimum energy considerations coupled with the varying surface topology of the patterned surface give rise to the nanocrystals, instead of a continuous ITO film. The ITO crystals are evidently ≈20 to ≈30 nm in size (Figure 2e,f). The white region in Figure 2f is SiO*_x_* which represents both native and air‐annealing‐associated oxide growth. Notably, during annealing in the air, oxygen diffuses into the Si sub‐surface, thereby oxidizing a few atomic layers of Si and creating a high‐quality interface between the *nc*‐ITO and underlying SiO*_x_*‐Si.^[^ ^22^ ^]^ This interfacial oxidation is examined in the sidewall region as shown in Figure 2g: ≈2 nm SiO*_x_* is grown between the *nc*‐ITO and nanostalagmite. The presented TEM image reveals that the native oxide attaches to the textured surface conformally. This suggests that the native oxide by exposing the sample in the air provides a conformal coating onto the entire surface—an important basis for avoiding complicated passivation techniques and exploring a facile way to get a high‐quality surface. Together with the surface band bending and the strong surface field, the chemical passivation effect of this native oxide layer contributes to a low photocarrier recombination rate. Further analysis of the *nc*‐ITO/nanostalagmite apex and in particular, the associated single crystal of ITO particle (Figure 2h) divulges the well‐organized ITO lattice orientation which is dominated by highly ordered In_2_O_3_ (222) planes. The conformality of the *nc*‐ITO contact is confirmed in Figure 2i, where highly ordered ITO crystals adhere to the nanostalagmite surface. It is noted that a sufficient conformality of the ITO coating is essential for effective photocarrier extraction, carrier transport within the ITO layer, and the electron injection in the contact region. In contrast to a circular distribution of diffraction points representative of polycrystallinity, the *nc*‐ITO on the nanostalagmite exhibits excellent single‐crystal‐like orientation; the linearly ordered diffraction points reveal both highly epitaxial ITO (yellow dotted line) and single‐crystalline Si (red dotted line) (Figure 2j). Additional HRTEM images are presented and elaborated upon in the Supporting Information (Figure S2).

### 2.2 Optoelectronic Characterization

**Figure 3:**
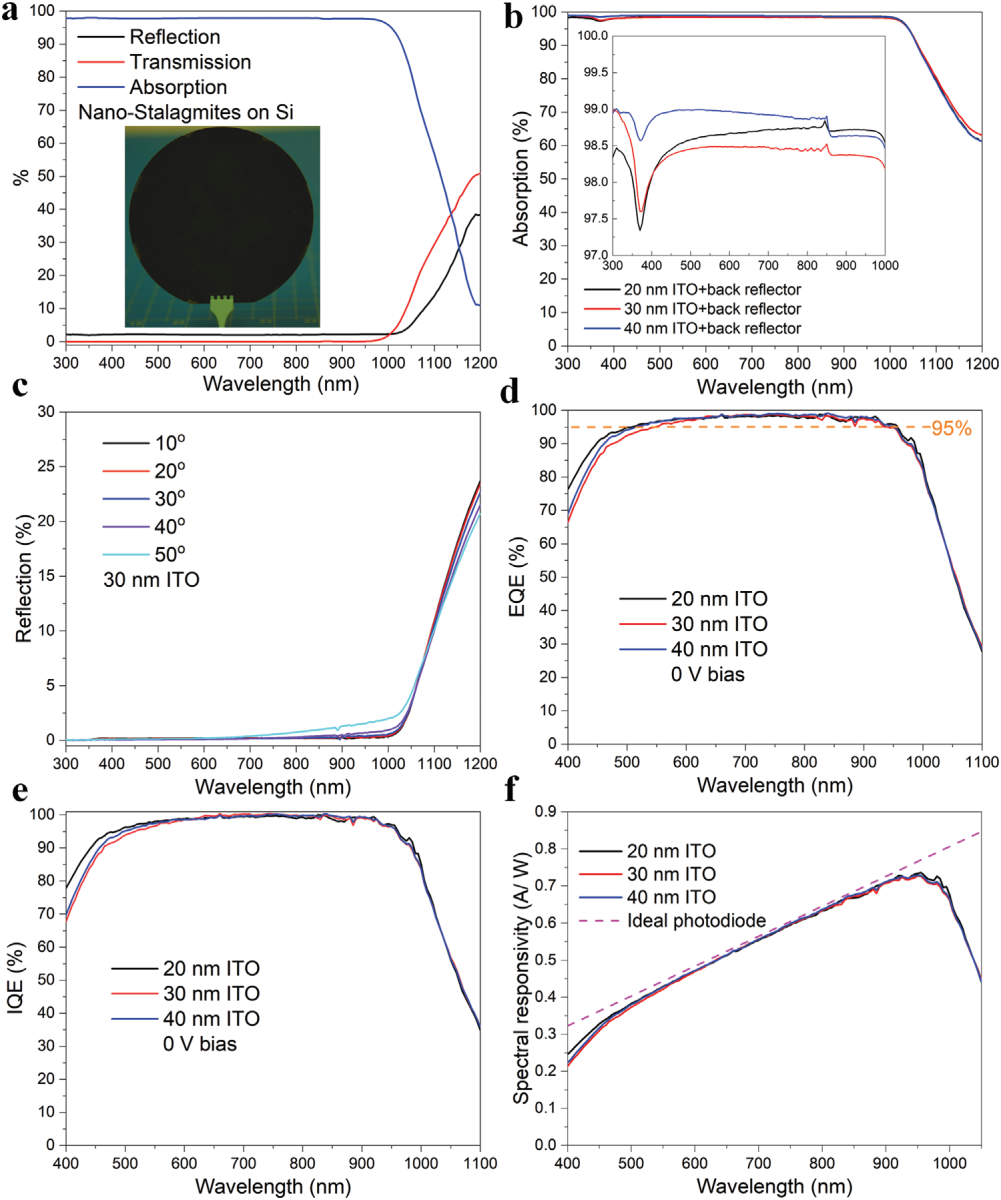
a) Spectral reflectance, transmittance, and absorptance of bare nanostalagmites on Si, including the inset showing a photograph of a 100 mm diameter *b*‐Si wafer. b) Absorptance of ITO/nanostalagmite/Al structures having different ITO thicknesses, including zoomed‐in inset. c) Angle‐dependent reflectance of 30 nm ITO/nanostalagmite/Al structure. d) EQE of nanostalagmite *b*‐Si photodiode having different ITO thicknesses. e) Calculated IQE of nanostalagmite *b*‐Si photodiodes. f) Calculated spectral responsivity of nanostalagmite *b*‐Si photodiodes, compared with the ideal responsivity of a photodiode.

The optical properties and the quantum efficiency are measured in **Figure** **3**. Figure 3a shows the reflection, transmission, and absorption of the bare (uncoated) nanostalagmite patterned silicon. An optical image of a 4 in. *b*‐Si wafer is shown as an inset in the figure, showing the viability of excellent large area wafer‐scale processing. High absorption of ≈97% on average is observed for wavelengths ranging from 300 to 1000 nm, a result of enhanced photon scattering by the dense distribution of the nanostalagmites. Compared with the optical spectra of the typical random pyramid texturing,^[^ ^24^, ^25^ ^]^ our nanostalagmite structure exhibits excellent absorption at short wavelengths, specifically in the 300–350 nm range, due to the pronounced sharp profile, as seen in Figure 2c; further, corresponding to the short absorption length where the photogeneration occurs over a span of a few nanometers into the silicon. The reflection of bare nanostalagmites in this work is compared with other nanostructures realized by MACE processes, such as needle‐shaped nanostructures,^[^ ^26^ ^]^ Si nanowire,^[^ ^27^ ^]^ nanostructured micropyramids,^[^ ^28^ ^]^ nanocone,^[^ ^29^ ^]^ nanospikes,^[^ ^30^ ^]^ and porous Si.^[^ ^31^ ^]^ The comparison is shown in Figure S3 in the Supporting Information. In general, nanostructures with low surface reflection are widely reported, and the ≈3% reflection attained in this work is moderately low among these nanostructures. We note that in the absence of a metallic back reflector, the transmission rate is negligible below 1000 nm wavelength. For example, ≈1.85% and ≈0.14% transmission rates are observed at 1000 and 960 nm, respectively. Below 960 nm wavelength, the transmission rate is almost 0% (below ≈0.1% or specifically of the order of ≈0.01% below 850 nm, which is comparable to the noise level of the UV‐Vis spectrometer), suggesting that nearly no light below 960 nm can transmit the Si absorber even without a metallic reflector on the backside. Upon deposition of the ITO thin film and ≈300 nm thick Al on the top and back surfaces, respectively, the average absorption increases to over ≈98.5% for *λ* spanning 300–1000 nm (Figure 3b, including the inset) principally due to the antireflective effect of the low index *nc*‐ITO. We calculate the optical absorption rate by measuring the reflection (see the Experimental Section); high absorption of over ≈98.5% from 300 to 960 nm happens to the *nc*‐ITO/*b*‐Si structure (no parasitic absorption from Al) considering the optical effects of ITO and *b*‐Si and that essentially no light transmits through Si even without a back‐reflector, as discussed above. The enhanced absorption at long wavelengths (≈61% at 1200 nm) is due to the free carrier absorption in Al thin film. Essentially omni‐directional low optical reflection is observed from the angle‐dependent measurements in Figure 3c where the nanostalagmite black Si exhibits low average reflectivity of ≈1% over a broadband of wavelengths for off‐normal illumination angles up to 50°. We note that this low off‐normal reflection is widely reported in nanostructured *b*‐Si.^[^ ^13^ ^]^ Examining the EQE results under 0 V bias in Figure 3d, the device with a 20 nm thin *nc*‐ITO contact exhibits an overall high quantum efficiency >≈90% across a broadband of wavelengths from 450 to 990 nm, and >≈95% from 500 to 960 nm wherein the average EQE exceeds ≈97% from 550 to 925 nm. For devices with ≈30 and ≈40 nm ITO, the average quantum efficiency is over ≈98% from 570 to 925 nm of wavelengths. The EQE of our nanostalagmite *b*‐Si device is compared with state‐of‐the‐art (SOA) Si PIN devices and leading‐edge Si heterojunction devices in Figure S4 in the Supporting Information,^[^ ^3^, ^4^, ^13^, ^14^, ^21^, ^32^ ^]^ and the dark current density and the fabrication technologies employed for each SOA device are compared with that of our device in Table S1 in the Supporting Information. Overall, the nanostalagmite *b*‐Si photodiode demonstrates high broadband quantum efficiency, especially at wavelengths above 500 nm, comparably low dark reverse current density, and is fabricated using a facile process. Moreover, at long wavelengths of 550–950 nm, our device demonstrates an EQE which is comparable to the reported advanced *b*‐Si PIN photodiode;^[^ ^13^ ^]^ suggesting that a single heterojunction photodiode can have the performance reaching that of the more complicated state‐of‐the‐art Si PIN devices. The repeatability of the high EQE results is shown in Figure S5 in the Supporting Information. It is worth noting that various factors do influence the small variability in the reported results; these include cable contacts during testing, inherent repeatability limit of the EQE test equipment, and unavoidable fabrication stochastics affecting the uniformity/variability error across a given wafer—all of which contribute to the small changes in the EQE measurements. Nevertheless, the statistical distribution (Figure S5b in the Supporting Information) of the data shows that the EQE lies within 97–99% for a range of devices, indicating excellent repeatability of this work. Analyzing the internal quantum efficiency (IQE) (Figure 3e), high IQE > ≈95% is observed for all samples from 520 to 960 nm. For devices with 20 nm ITO, near‐unity (>≈99%) average IQE is obtained from 635 to 920 nm, indicating that nearly‐all “far‐field” long wavelength generated photocarriers are collected owing to the pervasive built‐in electric field and commensurately enhanced photocarrier transport. For completeness, we present the device characteristics of a “near‐field device” wherein the *n*‐Si wafer has a resistivity of 2–10 ohm‐cm (≈10^15^ cm^−3^ doping level) (Figure S6 in the Supporting Information). Assessing the quantum efficiency from the perspective of loss analysis, we observe that there are drops in the IQE at short wavelengths (below 500 nm) and at long wavelengths (above 1000 nm). Comparing the EQE with devices made onto ≈10^15^ cm^−3^ doping and ≈10^12^ cm^−3^ doping Si substrates (Figure S7 in the Supporting Information), it is concluded that the ITO parasitic absorption and surface recombination are both critical factors for the drop in IQE at short wavelengths. A detailed analysis is presented in the Supporting Information. Specifically, the ≈20 nm ITO device exhibits a dramatic EQE/IQE increase at short wavelengths from ≈76% at 400 nm to ≈95% at 500 nm, indicating the benefit of a thin *nc*‐ITO layer where the free‐carrier‐associated absorption in ITO is markedly reduced. The IQE drop at long wavelengths is mainly due to the free carrier absorption in Al. From comparison of EQE of devices with varying doping levels (Figure S7 in the Supporting Information), it is concluded that the surface electric field plays an important role in photocarrier extraction. This also reflects that the chemical passivation effect of the native oxide is not eventually good, a partial reason for the drop in EQE at short wavelengths in Figure 3d. Further, it is worth mentioning that the field‐effect passivation has been widely reported to effectively passivate the *b*‐Si surface. For example, ALD Al_2_O_3_ has been demonstrated to provide excellent stable field passivation as an effective alternative to thermal oxide on the nanostructured *b*‐Si surface.^[^ ^33^, ^34^, ^35^ ^]^ And this surface field effect has been modeled to explain the low recombination rate of surface photocarriers, highlighting the importance of the surface electric field on photocarrier extraction.^[^ ^36^, ^37^ ^]^ We comparatively illustrate the mechanisms playing in two types of devices—“far‐field” and “near‐field” in the Supporting Information (Figure S8, Supporting Information). It is the confluence of a variety of factors that results in the near‐ideal quantum efficiency of the nanostalagmite *b*‐Si device. To sum up, the low‐aspect‐ratio nanostalagmite provides reasonably high absorption and easy‐to‐coat surface morphology. The coating of *nc*‐ITO increases the absorption up to 98.5–99% (*nc*‐ITO functioning as antireflection coating) over a broadband of wavelengths. This results in near‐100% absorption within the Si. Further, the *nc*‐ITO provides considerable surface electric field strength to reduce the photocarrier recombination and moreover, the SiO*_x_* provides a considerable surface chemical passivation effect to reduce the surface trap state densities. The field effect and the chemical passivation together contribute to excellent photocarrier extraction. Finally, the deep‐well depletion region adopted in this work ensures that most of the photogeneration principally occurs within the built‐in field region, resulting in effective photocarrier transport. A more detailed analysis of all the contributing factors that result in the realization of the high EQE is given in the Supporting Information. Further, we note the strategy proposed herein is not limited to *nc*‐ITO. In fact, this approach can be adopted for a range of possible contact materials in general, provided the contact material is optically thin (that allows near‐100% broadband light transmission), the work function of the contact material is selected so as to provide a sufficiently strong surface band bending (i.e., providing considerable surface electric field strength), and there is adequate surface chemical passivation (a material comparable to the native oxide of this work)—and as such lead to realization of near‐100% broadband EQE. The devices with different ITO thicknesses exhibit a near‐ideal spectral responsivity (SR) from 500 to 950 nm (calculated and compared with an ideal photodiode in Figure 3f). It has been reported for *b*‐Si PIN photodiodes with near ideal responsivity,^[^ ^16^, ^17^ ^]^ and it is also worth mentioning that there are already advanced commercial *b*‐Si PIN photodiodes with near ideal responsivity (e.g., PD1sM, PD5sMG from ElFys). Within the class of heterojunction photodiodes, near ideal responsivity has not been reported previously to the best of our knowledge. We present a comparison of the responsivity of our device with state‐of‐the‐art heterojunction photodiodes in Figure S9 in the Supporting Information.^[^ ^38^, ^39^, ^40^, ^41^, ^42^ ^]^

**Figure 4:**
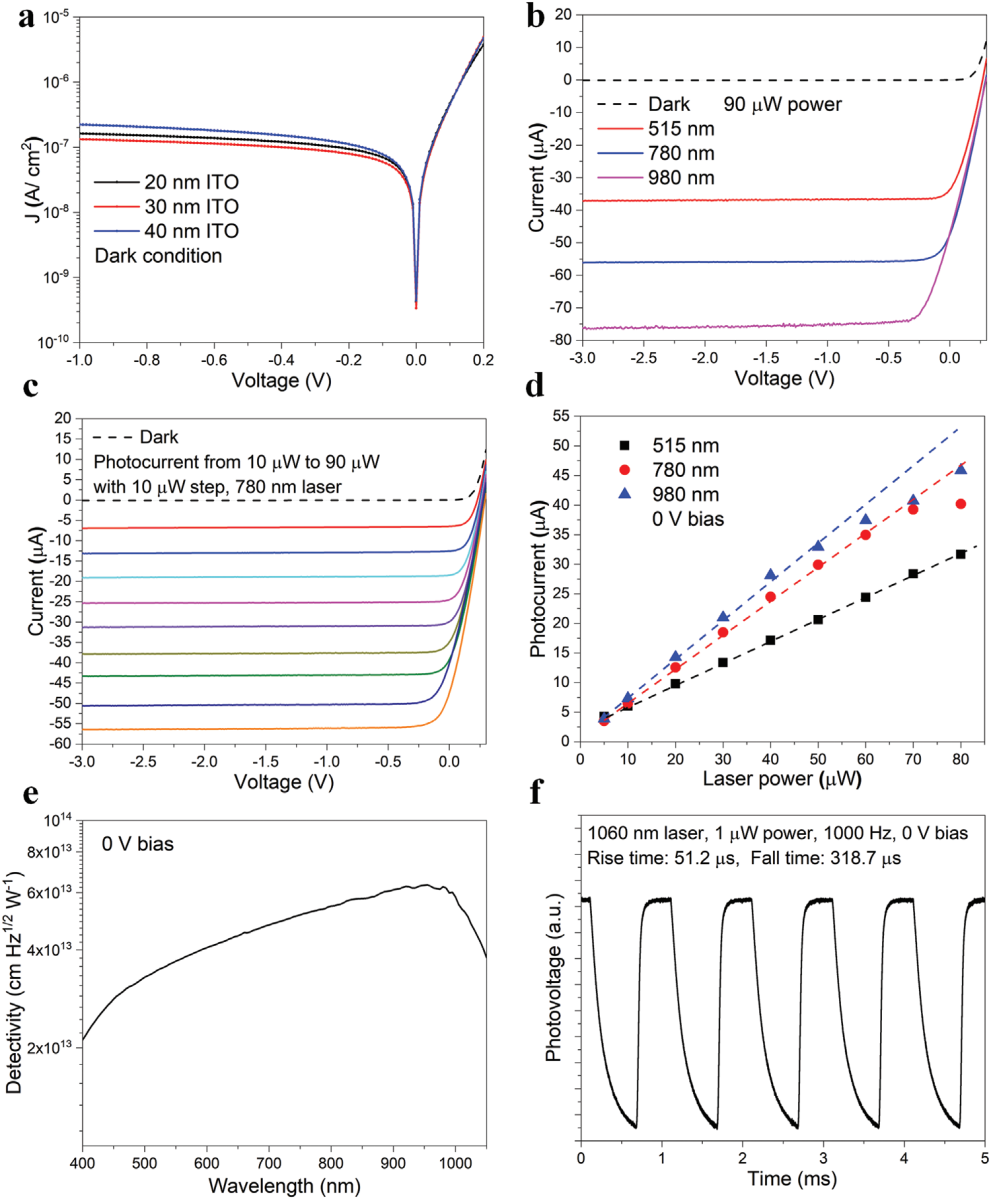
a) Dark current density–voltage (*J*–*V*) curves of devices with varying ITO thicknesses. b) Current–voltage (*I*–*V*) curves under laser illumination at various wavelengths (515, 780, and 980 nm) and constant power (90 µW). c) *I*–*V* curves measured under different laser powers (from 10 to 90 µW with 10 µW steps) using 780 nm wavelength laser. d) Photocurrent as a function of laser power under various wavelength illumination at 0 V bias. e) Specific detectivity at 0 V bias. f) Response speed at 1060 nm laser illumination, ≈1 µW power at 0 V bias.

Examining the photosensitivity of the devices, the current response under dark and various illumination conditions is shown in **Figure** **4**. The reverse dark current density for devices with various ITO thin film thicknesses is investigated in Figure 4a. All devices exhibit a low dark current density of ≈1.3 × 10^−7^ to ≈2.2 × 10^−7^ A cm^−2^ under a bias of −1 V, which is a factor of ≈2 lower than that of advanced ITO/*n*‐Si photodiodes.^[^ ^43^ ^]^ Moreover, it is noted that in comparison with the state‐of‐the‐art PIN photodiodes, the dark current density of our *b*‐Si Schottky photodiode is within one order of magnitude larger than that of the device reported by Savin et al.,^[^ ^13^ ^]^ whereas it is more than an order of magnitude lower than that of the PIN diode reported by Kim et al.^[^ ^14^ ^]^—remarkable performance metric considering that Schottky diodes naturally have higher reverse dark current densities than that of PIN diodes. Spectral photocurrent response for several wavelengths of laser spanning green to IR (515, 780, and 980 nm with a constant power of ≈90 µW) is presented in Figure 4b. Similar photocurrent trends are observed for all three wavelengths at biases ranging from 0 to −3 V. The photocurrents are ≈37.1, ≈56.1, and ≈66.8 µA for laser wavelengths of 515, 780, and 980 nm, respectively, at −3 V bias, corresponding to SR of ≈0.412, ≈0.623, and ≈0.742 A W^−1^, respectively. The photocurrent under 980 nm laser illumination begins to saturate at a reverse bias of ≈−0.33 V, while the photocurrent under 515 nm wavelength starts to saturate at the low reverse bias of ≈−0.1 V, indicating that an optimal electric field distribution within the *b*‐Si is required to collect all photogenerated carriers at a specific wavelength of interest. For example, for 515 nm wavelength, the absorption depth in Si is ≈0.685 µm; accordingly, a lower bias potential is adequate to generate a sufficiently strong near‐surface electric field to collect all the photogenerated carriers. Conversely, photons of 780 nm wavelength have an ≈8.92 µm absorption depth which in turn requires a higher reverse bias potential for complete photocarrier collection. To investigate the optical injection effects, we examine the linearity of photocurrent–voltage curves under various illumination powers (≈10 to ≈90 µW with 10 µW steps) using 780 nm laser (Figure 4c). Under small optical injection, the photocurrents saturate at 0 V bias, consistent with the near‐ideal EQE results in Figure 3d. These results demonstrate the viability of nanostalagmite *b*‐Si Schottky photodiodes to operate in a self‐biased mode for the detection of weak illumination signals. Under higher optical injection, a larger reverse bias is required for the photocurrent to reach the saturation level. For instance, the photocurrent saturates under a mere ≈−0.07 V bias under 50 µW illumination power, while the saturation threshold bias increases to ≈−0.3 V under ≈90 µW illumination—this is due to dramatically increased bulk Auger recombination and surface recombination losses under higher optical injection. The photocurrent on an exponential scale is presented in Figure S10 in the Supporting Information. For 90 µW laser power, at −0.1 V bias, the photocurrent to dark current ratio is ≈1.2 × 10^3^, ≈1.8 × 10^3^, and ≈2 × 10^3^ for 515, 780, and 980 nm wavelengths, respectively. At near 0 V bias, the photocurrent to dark current ratio is ≈2.05 × 10^5^, ≈2.88 × 10^5^, and ≈2.86 × 10^5^ for 515, 780, and 980 nm wavelengths, respectively. The high photo‐to‐dark current ratios illustrate the high sensitivity of the devices. Optoelectronic measurements for 515 and 980 nm wavelength under various laser powers are given in Figure S11 in the Supporting Information. Operating in self‐biased mode (0 V bias) and optical injection below ≈50 µW, the photocurrents at three wavelengths exhibit good linearity with increasing power (Figure 4d). Further, these results are consistent with the calculated SR (Figure 3f)—validating the near‐ideal response (also the near‐100% EQE) of the nanostalagmite self‐biased *b*‐Si device. The specific detectivity is calculated in Figure 4e, compared with the state‐of‐the‐art devices (summarized in Figure S12, in the Supporting Information). The device demonstrates high detectivity at 0 V bias of more than ≈2 × 10^13^ cm Hz^1/2^ W^−1^ (Jones) at 400–1050 nm wavelengths (≈0.63 × 10^14^ Jones peak at ≈955 nm). For completeness, it is noteworthy that today there are commercial *b*‐Si PIN photodiodes that achieve low noise equivalent power down to 10^−15^ W per √Hz (e.g., PD1sM, PD5sMG from ElFys). By way of comparison, we present the performance metrics of other (material‐wise) advanced heterojunction photodiodes, specifically comparing the high detectivity of these devices with our work: ≈10^12^ to ≈10^13^ Jones for organic photodiodes,^[^ ^44^, ^45^ ^]^ ≈10^12^ Jones for perovskite photodiodes,^[^ ^46^ ^]^ and ≈10^12^ to ≈10^13^ Jones for graphene/*n*‐Si photodiodes,^[^ ^4^, ^8^ ^]^ ≈10^10^ to ≈10^11^ Jones for type II Se/*n*‐Si heterojunction photodiode,^[^ ^47^ ^]^ and ≈10^11^ Jones for Si/perovskite photodiode.^[^ ^48^ ^]^ While it is recognized that each different material system will have its natural limit of specific detectivity, nevertheless, the high magnitude of specific detectivity of our single crystal Si devices is comparable to or markedly greater than that of these reported state‐of‐the‐art heterojunction photodiodes. We further note that our photodiode also shows a fast response for long wavelength generation (1060 nm illumination, 1 µW power): a mere ≈51 µs rise time is observed when the illumination is switched from “off” to “on” (Figure 4f). For completeness, it is noteworthy that the advanced *b*‐Si PIN photodiode has a nanosecond rise time.^[^ ^16^ ^]^ On the other hand, while various factors such as the incident wavelength and the light power may influence the measured rise time, the response speed demonstrated herein is nevertheless comparatively fast among the reported self‐biased Si heterojunction photodiodes: ≈140 µs rise time for MXene/Si heterojunction at 532 nm wavelength,^[^ ^1^ ^]^ ≈150 µs rise time for graphene/Si heterojunction at 633 nm wavelength,^[^ ^49^ ^]^ ≈20 µs rise time for n‐Si/p‐GaTe heterojunction,^[^ ^50^ ^]^ and ≈1 ms rise time for Si/perovskite heterojunction.^[^ ^51^ ^]^ The stability in the temporal response over a large time scale of 100 ms is illustrated in Figure S13 in the Supporting Information for 515 and 1060 nm wavelengths. Further, in addition to all the excellent benefits demonstrated, we discuss the potential shortcomings of the proposed device and possible strategies to further improve the device—a detailed discussion is given in the Supporting Information.

### 2.3 Theoretical Simulation and *b*‐Si Integrated Heart Rate Monitor

**Figure 5:**
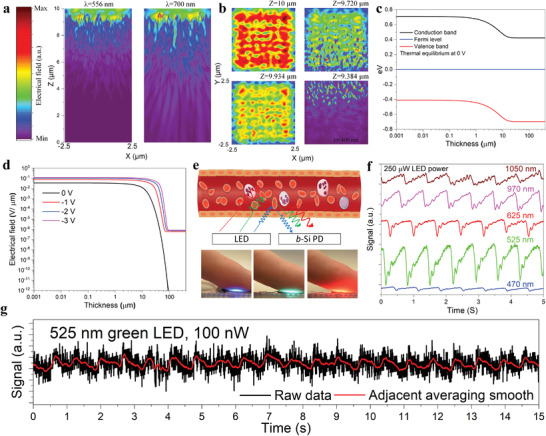
a) Simulated electric field distribution for the *X*–*Z* cross‐section under illumination wavelengths of 556 and 700 nm. The dimension for the simulated area is 5 µm (*X*) × 10 µm (*Z*). b) Simulated electric field distribution for *X*–*Y* cross‐section at various values of height (*Z*), under 400 nm wavelength incident light. The dimension for the simulated area is 5 µm (*X*) × 5 µm (*Y*). All figures in (a) and (b) have the same scale bar for the electric field. c) Simulated device energy band diagram at thermal equilibrium. d) Simulated electric field under different reverse biases across the thickness of the wafer. e) Illustration of the heart rate sensing mechanism where the reflected light modulates with the pulsing blood flow. f) Heart rate signal under blue (470 nm), green (525 nm), red (625 nm), NIR (970 and 1050 nm) LED illuminations (250 µW constant power). g) Heart rate signal under faint illumination (green LED, 100 nW). The smooth red curve represents a 30 point moving average.

3D finite‐difference time‐domain (FDTD) modeling is conducted to investigate the optical generation in these low‐aspect‐ratio nanostructures. Si thickness direction (vertically up) is denoted as the *Z*‐axis, and the scanning area direction is denoted as the *X* and *Y* axes. The tip point of the highest nanostalagmite is defined to be the top surface (*Z* = 10 µm). The *X*–*Z* cross‐sectional electric field distribution is shown in **Figure** **5**a. For incident illumination of 556 nm wavelength, most of the optical confinement is within ≈2.5 µm depth from the Si surface, and this depth increases to ≈5 µm for 700 nm incident light, given the larger absorption depth for Si at longer wavelengths. The electric field distributions for both 556 and 700 nm illumination indicate an excellent antireflection effect of the low‐aspect‐ratio nanostalagmites, where nearly‐all photons are redirected into the Si absorber due to the enhanced scattering effect. This is in good agreement with the observed low reflection (less than ≈3%) of the bare nanostructures (Figure 3a). The *X*–*Y* cross‐sectional electric field distribution for various *Z* values is presented in Figure 5b under the illumination wavelength of 400 nm. Since Si has an absorption depth of ≈82.25 nm for 400 nm wavelength, most of the incident photons are confined in the tip/middle region of the nanostalagmites (see the electric field distribution when *Z* is 10 and 9.934 µm). The nanostructures with smaller tip heights also contribute to the antireflection effect as observed when *Z* is 9.934 µm. The photon field begins to diminish at *Z* = 9.72 µm after ≈280 nm of downward travel distance from the top surface; and the electric field becomes zero for most of the regions when *Z* is 9.384 µm, indicating that nearly all the photons are absorbed within the nanostalagmites before being redirected into bulk Si absorber. This explains the quantum efficiency loss at short wavelengths observed in Figure 3d (≈76.32% EQE at 400 nm for devices with 20 nm ITO thickness). In addition to free carrier absorption in ITO thin films, the surface coverage (meaning the conformality of contact) and the surface recombination contribute to the effective photocarrier collection as well. For photogeneration at long wavelengths >600 nm, this effect is completely overcome by drift‐dominated photocarrier transport and nearly all photons are absorbed in the deep region of bulk Si, leading to a facilely fabricated device achieving near‐100% EQE. 1D finite element analysis (FEA) modeling is carried out to better understand the device performance in Figure 5c,d. The Schottky barrier height of the device is measured in Figure S14 in the Supporting Information. The modeled energy band diagram at thermal equilibrium (0 V) is plotted as a function of device thickness (≈400 µm) in Figure 5c under no illumination. A depletion region width of ≈40 µm is obtained, illustrating the built‐in electric field matching with the optical generation in Figure 5a. These results also confirm the mitigation of the electron tunneling effect under reverse bias (Figure 4b), and the saturation of the photocurrent. The electric field across the entire wafer thickness is obtained by solving Poisson's equation (Figure 5d). The peak electric field at the interface is of the order of ≈0.1 V µm^−1^ for all input voltages. This naturally induced surface field effect of a Schottky junction contributes to a high‐quality interface with mitigated surface recombination for photocarriers. At thermal equilibrium, the electric field extends into the Si absorber to a depth of a hundred µm (≈10^−12^ V µm^−1^ at ≈100 µm at 0 V bias). These results confirm the near‐ideal quantum efficiency and rapid temporal response especially for direct band‐to‐band transitions associated with photon absorption at long depths. The fact that most of the photocarriers (from 500 to 960 nm wavelength) are generated within the built‐in electric field region is critical for effective carrier transport and collection. For example, most of the photogeneration at 700 nm wavelength is confined within the 10 µm distance from the top surface, as illustrated in Figure 5a. This partially explains the 98% EQE of the device: photocarriers can be swept toward the contact region (top contact) effectively due to the field effect on photocarriers generated in both the depletion region and the tailing electric field region (as observed in the simulated data in Figure 5d). For comparison, the devices with a narrow depletion region (prepared on Si substrate with dopant density of ≈10^15^ cm^−3^, Figure S7 in the Supporting Information) show ≈90% EQE at 900 nm, indicating that a significant fraction of photocarriers generated at this wavelength recombine at the unoptimized backside or recombine during the transport process (i.e., they are generated in the neural region). Indeed, we can explore this for a range of photogeneration scenarios. For instance, an absorber designed for photogeneration that principally happens in the near‐surface region, the device would be configured with a strong near‐top surface field, as shown in Figure S6 in the Supporting Information. Under larger reverse bias potentials (−1, −2, and −3 V), the strong electric field region (field strength greater than ≈10^−6^ V µm^−1^) extends to a hundred µm, with a uniformly distributed electric field from the surface to ≈40 µm depth, thus enabling a higher collection efficiency under high optical injection (confirmed in Figure S11d, in the Supporting Information). The modeled energy band diagram under −3 V bias and carrier concentrations at various reverse potentials in the device are given in Figure S14 in the Supporting Information.

A nanostalagmite *b*‐Si photodiode is integrated into a heart rate monitor driven by faint light, as demonstrated. The measurement mechanism is shown in Figure 5e, where the light from a light‐emitting diode (LED) source is reflected by human blood. Variations in the reflected light correspond to changes in optical scattering by the pulsing blood and thus the heart rate is recorded by the black Si photodiode. The measurement process is described in detail in the Experimental Section. A heart rate sensor driven by multiwavelength photons (as shown in Figure 5e, where the light at different wavelengths impinges on the test finger) has the advantage of providing a more accurate characterization of the heart pulse as well as additional blood vitals. For example, accurate multispectral data can be correlated to the presence of various components (such as lactate, potassium, red blood cells, and glucose) in humans amenable for health monitoring as well as real‐time athletic performance assessment. The optical photo for the heart rate sensor set up is given in Figure S15 in the Supporting Information. For a constant illumination power (≈250 µW), the heart rate test results are shown in Figure 5f, for LED illumination at 470, 525, 625, 970, and 1050 nm wavelengths. A high signal‐to‐noise ratio is observed for all wavelengths, exhibiting excellent signal detection by the *b*‐Si photodiode over a broadband of wavelengths. Notably, the sensor reveals high sensitivity under NIR (1050 nm) illumination, where the optical absorption by the blood is relatively low. Further, the *b*‐Si photodiode operating under no externally applied bias (no‐power‐consumption mode) is able to detect very faint light signals (Figure 5g). With the green light, the heart rate monitor exhibits sensitivity with a mere 100 nW of illumination power. This power level (to the best of our knowledge, the lowest reported LED driving power for a heart rate sensor) is >1.7 × 10^5^ times lower than that in a commercial heart rate sensor (Maxim Max30101, ≈17.2 mW power for green LED). Heart rate measurements under 1 µW and 200 nW for a green LED and under 500 nW for 970 nm NIR LED are examined in Figure S16 in the Supporting Information. These “reduction to practice” experiments illustrate the potential to apply the near‐ideal performance of the nanostalagmite *b*‐Si photodiode for multifunctional applications where low power consumption and high sensitivity are paramount. A detailed discussion of the advantages of the demonstrated heart rate sensor is presented in the Supporting Information, and a comparison with other reported heart rate sensors is summarized in Table S2 in the Supporting Information.^[^ ^44^, ^52^, ^53^, ^54^ ^]^

## 3 Conclusion

A self‐biased nanostructured *b*‐Si heterojunction photodiode is demonstrated herein. Low‐aspect‐ratio nanostructures are realized that carefully balance optics and surface geometry characteristics. Under no external bias, the devices exhibit near‐complete quantum efficiency and near‐ideal spectral response over a broadband of wavelengths. High detectivity and fast photocurrent response are demonstrated. Further, an extremely sensitive heart rate monitor driven by faint light is demonstrated, showing great potential for nanostalagmite *b*‐Si heterojunction photodiode for high performance and low power consumption applications. It is expected that the results of this research will pave the way in the realization of ideal *b*‐Si integrated optoelectronic applications at a low cost.

## 4 Experimental Section

### Nanostalagmite Fabrication

The fabrication commenced with prime grade Si wafers (n type phosphorus doped, ≈3000 ohm‐cm as determined by a four‐point probe, 400 µm thickness). A metal‐assisted chemical etching process was explored to fabricate the nanostalagmites. A unique combination of Au nanoparticle deposition, wet chemical etching, and polishing processes was utilized. The synthesis steps started with the deposition of ≈3 nm Au nanoparticles onto Si wafer by using electron beam evaporation (Kurt J. Lesker). The native Si oxide layer was not etched prior to the deposition. The chamber was pumped with pressure below 10^−7^ Torr before deposition. The deposition rate of Au was held at ≈0.1 Å s^−1^, which was a result of process optimization to achieve an appropriate Au nanoparticle distribution. Subsequently, a common etching solution comprising hydrogen peroxide (H_2_O_2_, 30%), hydrofluoric acid (HF, 49%), and deionized (DI) water was used to etch the Si. The concentration and the etching time were optimized as follows: samples were dipped in a mixture of etching solution (H_2_O_2_ (30%), HF (49%), and DI water mixed in the proportion of 5:2:10) for 2 min at room temperature. After that, the samples were dipped in gold etchant (651818, Sigma‐Aldrich) to remove the remaining Au, followed by RCA cleaning to slightly polish the nanostructures and to remove residual contamination off the patterned surface. Unlike standard RCA cleaning at 75°–80° of temperature, the temperature was reduced to avoid over‐polishing of the nanostructures. H_2_O, NH_4_OH, and H_2_O_2_, mixed in the proportion of 12:1:2, were used to clean the samples at 40 °C for 10 min. Then, H_2_O, HCl, and H_2_O_2_ mixed in the proportion of 6:1:1 were used to clean the samples at 60 °C for 10 min. After cleaning, the samples were dipped in buffered oxide etchant (6:1) for 2 min to remove the silicon oxide layer.

### Device Fabrication

After fabrication of the nanostalagmites, the samples were then exposed to air in order to grow the native SiO*_x_*. Then the samples were loaded into the sputter‐deposition chamber (Kurt J. Lesker) for the deposition of *nc*‐ITO. A shadow mask was used to define the contact region. After post‐annealing treatment of *nc*‐ITO thin films, ≈300 nm Al was sputter‐deposited on the backside of the wafer, thus serving as both an ohmic contact and an optical back reflector which completed the device fabrication.

### nc‐ITO Deposition

The developed deposition recipe is as follows: the chamber was pumped down to a pressure below ≈10^−6^ Torr prior to deposition. A standard 3 in. ITO target (In_2_O_3_:SnO_2_ = 90%:10%) was used as the sputtering source. Presputtering was conducted before every formal sputtering for making devices. The flow of 20 sccm Ar/O_2_ (2% O_2_) was established for the deposition process. ≈100 W power and ≈2.5 mTorr pressure were set to achieve a deposition rate of ≈1.5 nm min^−1^, which was monitored by a quartz sensor. The samples were then annealed at 400 °C for 30 min in air.

### Surface Morphology Characterization

HRSEM imaging was performed using the Hitachi S‐5200/SU‐8230 ultra‐high resolution SEMs. AFM imaging was conducted using a Dimension Icon AFM (Bruker). All scans were performed using the ScanAsyst analysis mode and using a Bruker ScanAsyst‐Air probe. The AFM results were further analyzed using the software NanoScope Analysis. The HRTEM images were obtained with a Hitachi HF‐3300 environmental TEM, while the EDS profiles were acquired using a Bruker 6 | 60 detector integrated within the same TEM system.

### Optical Characterization

The spectral optical transmittance *T* and reflectance *R* profiles were measured using a UV‐Vis Spectrophotometer (Perkin Elmer Lamda 1050). The absorptance *A* of bare nanostalagmite on Si was then deduced by the following relationship

(1) A=100%−T−R

For *nc*‐ITO/nanostalagmite on Si/Al devices, there was no light transmission due to the presence of the Al back reflector, hence *A* was calculated by the relation

(2) A=100%−R

### Optoelectronic Characterization

The EQE measurements were conducted by a commercially available QE‐R system from Enlitech (with greater than ≈99.5% repeatability). A Xenon lamp was installed in the system. The light was then directed into a monochromator for single wavelength beam output. The wavelength step was set to 5 nm to obtain accurate measurements. The single wavelength light beam was directed onto the active area of the device. The illumination was modulated via a chopper (165 Hz) which was connected to a lock‐in amplifier, thereby ensuring that the device output photovoltage signal frequency was synchronized for accurate signal recording. The temperature of the measurement state was set to 25 °C using a temperature controller. The averaging sampling rate during data acquisition was set to 15, while the allowed noise range was set to 5E‐4 for precise measurements. Prior to the EQE measurement, the system was calibrated against two reference photodetectors: Si photodetector from 300 to 1100 nm (Enlitech Model RC‐S103011‐E) and Ge photodetector from 1100 to 1200 nm (Enlitech Model RC‐G108018‐E). The signal‐to‐noise ratio of the two reference photodetectors is given in Figure S17 in the Supporting Information. The EQE repeatability of separate devices is shown in Figure S5 in the Supporting Information. The IQE was calculated by the following equation

(3) IQE=EQE/Absorption

where the absorption results in Figure 3b were utilized. The spectral responsivity *R_*λ*_* was determined by

(4) $$R_{\lambda }=\mathrm{EQE}\times \lambda \times \frac{e}{hc}$$

where *λ* is the wavelength of photons, *e* is the elementary charge, *h* is Plank's constant, and *c* is the vacuum light speed.

Current–voltage (*I–V*), capacitance–voltage (*C–V*), and temporal response with on/off illumination were measured using a Keithley 4200 semiconductor analyzer. Diode lasers (Thorlabs) of different wavelengths (515, 780, and 980 nm) were properly aligned and focused onto the device region. The measured photocurrents by using the Keithley 4200 semiconductor analyzer (0.1 fA rated noise measurement with two remote amplifiers (4225‐RPM) for ultra‐low current measurement) also confirmed the high EQE results. The incident laser beam was calibrated by a sensitive optical power meter (Thorlabs PM200, with a S120C photodiode power sensor for power levels down to nanowatts). The photocurrents (linear region) in Figure 4d and the correspondingly calculated responsivity were consistent with the measured high EQE results as per the relationship given in Equation (4).

The photocurrent *I*_ph_ was calculated by

(5) $$I_{\mathrm{ph}}=I_{\mathrm{light}}-I_{\mathrm{dark}}$$

where *I*_light_ is the current under illumination and *I*_dark_ is the dark current.

The specific detectivity D* was calculated by

(6) $$D^{*}=\frac{R_{\lambda }\sqrt{S}}{\sqrt{2eI_{\mathrm{dark}}}}$$

where *S* is the effective area of the photodiode.

For temporal response measurements, fiber‐coupled diode lasers at 515 and 1060 nm wavelengths (Thorlabs LP515‐SF3 and Qphotonics QFBGLD‐1060‐10BTF) were mounted into laser mounts, which were connected to a laser driver (Thorlabs LDC 210C) and a temperature controller (Thorlabs TED 200C). A function generator (Hewlett Packard 33120A) was connected to the laser driver, to impress a square signal to modulate the laser frequency at 1000 Hz. The photovoltage of the devices was recorded by a digital oscilloscope (Tektronix MDO3024).

### Finite Element Analysis (FEA) Modeling

To show the built‐in electric field across the whole wafer thickness (400 µm) and to reduce the overall simulation load, 1D semiconductor physics module was taken in the COMSOL Mutilphysics simulation software to determine the band diagram, electric field, and carrier concentration in the device. Schottky barrier height of ≈0.71 eV was used to define the contact. The resistivity of 3000 ohm‐cm, corresponding to ≈1.472E12 cm^−3^ electron concentration, was used to define the Si doping level. Poisson's equation was solved across the thickness of the device.

### Finite‐Difference Time‐Domain (FDTD) Modeling

3D FDTD optical simulations were carried out using commercial software (Lumerical FDTD). The AFM results (5 µm by 5 µm scanning area as indicated in Figure 1j) were imported as the representative nanostructure surface profile. Considering that for wavelengths <700 nm, most of the photons were absorbed in the “near‐surface” region, 10 µm thickness Si was utilized to reduce the computational load. 3D FDTD was built for the entire 5 µm × 5 µm × 10 µm area with proper meshing. A plane wave source was used for normal illumination. For different wavelength scans, a 2D monitor was used for *X*–*Z* cross‐sectional electric field simulation (Figure 5a), while a 3D monitor was used for different depth scans (*X*–*Y* cross‐sectional field distribution) at a fixed 400 nm wavelength (Figure 5b).

### Heart Rate Monitor

The informed written consent from volunteers was collected. According to the Ethical Conduct for Research Involving Humans—TCPS 2 Article 2.5, it was confirmed that there was no need for institutional review board approval for the heart rate tests, which fell under the purview of quality improvement studies and experimental testing for assessment purposes, and that the volunteers participated in a simple index finger contact measurement without any physical modification nor invasive procedure. The facile heart rate monitoring set‐up is shown in Figure S15 in the Supporting Information. The surface‐mounted LEDs at different wavelengths and the black Si photodiode were mounted contiguously onto a breadboard. A DC power supply (Agilent E3647A) with a precise 10 mV voltage control was utilized to drive the LED to the required power level. The output of the LED was calibrated against a sensitive optical power meter (Thorlabs PM200, with a S120C photodiode power sensor). The heart rate monitoring experiments were carried out in a dark room (with 5–10 nW environmental light noise due to light from the proximal digital oscilloscope and lock‐in amplifier screen). The S120C photodiode sensor enveloped and collected all the LED lights during calibration. No voltage bias was applied to the *b*‐Si photodiode and thus all photocurrent signals were obtained in a self‐biased operational mode. The light from the LEDs was redirected (reflected) by the volunteer's test finger (blood) into the photodiode. The photodiode was connected to a signal amplifier (EG&G Princeton Applied Research, Model 5302), using a low pass filter (<10 Hz). The output voltage was recorded by a digital oscilloscope (Tektronix MDO3024). For heart rate monitoring using a 100 nW LED, 30 point moving average was used to obtain the smooth red curve shown in Figure 5g.

### Statistical Analysis

All experiments were conducted for more than three times to confirm the reproducibility of the results. A fill‐area‐under‐curve processing was performed for the EDS data in Figure 2b using a software OriginPro 2018. The transient photovoltage results (Figure 4f and Figure S13, Supporting Information) were normalized to the maximum photovoltage. A 30 point adjacent average smooth was processed as red curve in Figure 5g, where the directly measured raw data (black curve) were also showed. The adjacent average smooth was conducted by a software OriginPro 2018. All the rest of results were shown as directly measured without preprocessing. The statistical data shown in Figure 1k were performed by a software NanoScope Analysis. Five separate devices were measured to show the statistical data in Figure S5b in the Supporting Information, and the statistical analysis was conducted by a software OriginPro 2018.

## Conflict of Interest

The authors declare no conflict of interest.

## Supporting information

Supporting Information

## Data Availability

The data that support the findings of this study are available from the corresponding author upon reasonable request.
