# Supplementary material for: Facilely Achieved Self‐Biased Black Silicon Heterojunction Photodiode with Broadband Quantum Efficiency Approaching 100%
Source: Adv Sci (Weinh). 2022 Oct 17;9(33):2203234. doi: 10.1002/advs.202203234 (PMC9685453; doi:10.1002/advs.202203234)
Supplement: Supplementary file 1 — Supporting Information [file ADVS-9-2203234-s001.pdf]

ADVANCED SCIENCE
Open Access

Supporting Information

for *Adv. Sci.*, DOI 10.1002/advs.202203234

Facilely Achieved Self-Biased Black Silicon Heterojunction Photodiode with Broadband Quantum Efficiency Approaching 100%

*Yibo Zhang, Joel Y. Y. Loh and Nazir P. Kherani**

Supporting Information

Facilely Achieved Self-Biased Black Silicon Heterojunction Photodiode with Broadband Quantum Efficiency Approaching 100%

*Yibo Zhang, Joel Y. Y. Loh, and Nazir P. Kherani**

1. SEM images for nanostructures before AuNP removal

To better explain the *b*-Si photodiode fabrication process, SEM images at different magnification rates for samples prior to the removal of gold nanoparticles (AuNP) are shown in **Figure S1**. It is observed that the etching process creates a nano-tree like porous structure with a period of ~300 nm (**Fig. S1a**). Highly uniform nanostructures are obtained with the AuNP deposition and follow-on metal-assisted etching process. In these images, AuNPs are left embedded in the nanostructures, and a rough surface is observed. This rough surface which introduces a large number of defects and a large effective area are the principal challenges achieving high quantum efficiency in all *b*-Si devices due to the enhanced trap-assisted photocarrier recombination loss. As mentioned in the manuscript, the RCA cleaning process slightly polishes the structures, and it is asserted that this polishing process does not dramatically alter the overall absorption but instead yields a less rough surface amenable to optimizing the follow-on contact deposition process, thereby contributing to high quantum efficiency.

2. HRTEM images of nano-stalagmites

The HRTEM images for *nc*-ITO/ nano-stalagmites illustrate an array of nano-stalagmites (**Figures S2a** and **b**). The height of the nano-stalagmites lies between ~500 nm to ~600 nm, in keeping with the statistical distribution shown in the AFM images (**Figure 1m** in the manuscript). **Figure S2b** confirms the non-ideal conformal distribution of *nc*-ITO on the nanostructured

surface, implying the need for a proper electric field profile whereby the desired high performing *b*-Si device is achieved. The HRTEM image corresponding to the diffraction pattern (**Figure 2j**) is shown in **Figure S2c**. Examining the large scale (100 nm scale bar) image where *nc*-ITO are attached to the surface of the nano-stalagmites, the corresponding diffraction pattern of **Figure 2j** establishes that the *nc*-ITO is oriented highly-epitaxially on the nano-stalagmites and over large areas. Further, the single-crystal *nc*-ITO exhibit excellent lattice matching with the *n*-Si (**Figure 2j**, illustrating the dotted line diffraction patterns for both Si and *nc*-ITO lattice). The diffraction pattern for *n*-Si is shown in **Figure S2d**.

3. Comparing the reflection of these nano-stalagmites in this work with the reported nanostructured b-Si fabricated by the metal assisted etching process.

The reflection of the nano-stalagmites in this work is compared with other nanostructures fabricated by metal-assisted etching process in **Figure S3**.[1-6] Among these nanostructures, the reflection in this work is moderately low. Optical impendence matching is the primary reason explaining the low reflection of nanostructures. Optical impedance matching is the suppression of reflection losses (Fresnel reflection) at interfaces between two media which is achieved by creating a gradual transition in the optical index upon traversing from one medium into another. In the present work, the nano-stalagmite patterning of the silicon surface provides a smooth transition from air to silicon.

4. Comparing the performance of nano-stalagmite *b*-Si photodiode with state-of-the-art photodiodes vis-à-vis dark current density, EQE, and complexity/simplicity of fabrication methods

We present a detailed comparison of the nano-stalagmite *b*-Si photodiode with the state-of-the-art photodiodes vis-à-vis EQE, SR, dark current density, the morphology of the light trapping/anti-reflection structure and fabrication technology in **Figure S4** and **Table S1**.[7-12] We first consider state-of-the-art heterojunction photodiodes (see **Figure S4)**.[9-12] Poly (3,4-ethylenedioxythiophene): poly (styrenesulfonate) (PEDOT: PSS), Graphene and solution-processed Silver nanowires (AgNW) based devices, integrated on Si, exhibit a relatively lower

EQE of 50% to 80% for broadband of wavelengths. Further, some of these devices require additional reverse potential to achieve higher collection efficiency.[9] On the other hand, the EQE performance of this work is comparable with the state-of-the-art PIN photodiodes.[7] However, we utilize direct PVD (physical vapor deposition) based single-contact device construct, in contrast to relatively complicated multi-process fabrication processes (**Table S1**) such as reactive ion etching, high-temperature annealing for dopant diffusion to fabricate PIN junctions, and complicated ALD/CVD processes to passivate the nanostructures.[7] Specifically, our device requires a simple PVD and solution etching process to create elegant *b*-Si nanostructures, a simple PVD process to make two contacts (Schottky contact on top and ohmic contact on the rear), and a low-temperature air-annealing process.

In sum, the performance of our devices far exceeds that of state-of-the-art *b*-Si heterojunction photodiodes and are comparable with or even remarkably outperforms some PIN based *b*-Si photodiodes while requiring simple fabrication, realized by well-thought-out design elements.

5. Repeatability test for EQE at various devices

The EQE results are repeated by measuring different devices in **Figure S5**. **Fig. S5a** shows the full spectrum EQE test for three different photodiodes. **Fig. S5b** gives EQE statistics at different wavelengths of 600 nm, 700 nm, 800 nm and 900 nm.

6. Performance characterization for devices fabricated with 2-10 Ω-cm resistivity *n*-Si

In order to understand the influence of electric field distribution on device performance, 2-10 Ω-cm *n*-Si wafers are utilized to fabricate the nano-stalagmite *b*-Si photodiode (**Figure S6**). The photodiode exhibits broadband high quantum efficiency of greater than ~90% from 400 nm to 900 nm and greater than ~95% from 450 nm to 850 nm (**Figures S6a, b**). Considering that the higher doping level of the wafer results in a narrower depletion region and a stronger built-in electric field, we observe a slightly higher EQE at short wavelengths. However, the EQE drops below 90% at wavelengths below 900 nm compared to devices designed for the wide electric field that show EQE of >90% from ~450 nm to 990 nm. The spectral responsivity for 2-10 ohm-cm samples with

various ITO thicknesses are presented in **Fig. S6c**. Linear and near ideal SR is observed from 500 nm to 900 nm, which is in good agreement with the results presented in **Fig. 3f** in the manuscript, however, the wavelength at which EQE begins to drop moves to 900 nm due to carrier recombination loss for long wavelength photogeneration. For devices made on lower resistivity Si (2-10 Ω-cm, 10^{15} cm^{-3}), we observe that the reverse dark current increases dramatically with larger reverse bias (**Fig. S6d**), which is contrary to the almost-constant reverse current observed for devices made on 10^{12} cm^{-3} Si (**Fig. 4a**). The reverse dark current density is observed to increase from ~10^{-8} A/ cm^2 at 0 V to ~10^{-5} A/ cm^2 at -3 V (**Fig. S6d**). The reverse dark current density also increases with a thicker ITO film: the ~40 nm thickness ITO device shows the highest leakage current under reverse bias. In contrast, the J-V results for devices made on 10^{12} cm^{-3} Si (**Fig. 4a**) are less dependent on the ITO thickness. A number of factors are likely at play giving rise to the observed variation in leakage current among the devices: dissimilar energy band alignments between ITO and *n*-Si, variations in the depletion region width and correspondingly different geometry-dependent carrier tunnelling processes (direct tunnelling or trap-assisted tunnelling), and difference in bulk carrier generation

7. Comparing the EQE between devices with/without nanostructures and devices with varying Si doping concentrations.

Here we present the EQE results for the planar Si and the *b*-Si devices in **Figure S7**. We also include the EQE results for the planar Si device with 10^{15} cm^{-3} doping.[13] The graph below clearly shows that the performance achieved from our low-aspect ratio facile fabricated *b*-Si device far exceeds planar Si. Also, we highlight the fact that the present study is clearly part of a continuous effort in the field to push forward device performance toward 100% broadband EQE. For planar Si (~10^{15} cm^{-3} doping) with ITO coating, the EQE at 900 nm wavelength is ~71% EQE (color cyan).[13] With the present work, the EQE improves to ~79% for a similar planar Si device with a lower doping level (color green); and is further improved to ~91% with well-designed textured Si devices with 10^{15} cm^{-3} doping level. The ultimate optimization of the devices (with doping level of 10^{12} cm^{-3}) leads to performance improvement in the EQE value to ~98% (color black, red and blue). Moreover, this high EQE of 98% is not only for a single wavelength, but realized for wavelengths ranging from 550 nm to 950 nm.

8. Field distribution comparison between devices fabricated with 2-10 ohm-cm and 3000 ohm-cm resistivities

To elucidate the importance of field profile in realizing high performance nano-stalagmite b-Si photodiodes, schematic diagrams of devices made of 2-10 ohm-cm and 3000 ohm-cm are illustrated in **Figure S8**. As simulated in **Figure 5d** in the manuscript, we observe that in addition to a strong built-in electric field across the depletion region, there exists a weak field region where the magnitude of the field strength is below 0.01 V/ µm and extends some 100 µm into the silicon wafer (**Figure S8a**). This correlates well with the photocarrier generation due to long wavelength light (from 750 nm to 950 nm) and thus explains the excellent unity IQE. The device made of 2-10 ohm-cm silicon, however, creates a strong field region near the surface and thus results in a very large neutral region where photocarrier transport is diffusion limited (**Fig. S8b**). Not surprisingly, higher EQE in these devices occurs at short wavelengths (~95% at 450 nm) and then drops off rapidly at long wavelengths (~83% at 950 nm, in contrast to ~96% (**Figure 3d** in the manuscript) for the wide-field designed device).

9. Comparing the spectral responsivity in this work with the state-of-the-art reported heterojunction *b*-Si photodiodes

The spectral responsivity in this work is compared with the reported heterojunction *b*-Si photodiodes in **Figure S9**.[14-18] To the best of our knowledge, the near-ideal spectral responsivity achieved in this work has not been reported previously among the set of all Si heterojunction photodiodes.

10. Detailed discussion regarding the mechanisms underlying the reported high EQE of the present work

First, the tapered nanostructures fabricated in this work provide a reasonably low reflection (3% reflection, effectively corresponding to 97% absorption, **Figure 3a**) over the spectrum of wavelengths, as well as an easy-to-coat surface morphology. The easy-to-coat surface makes it

possible that a facile physical vapor deposition process can produce thin films of high-quality coating (*nc*-ITO) on the surface. Due to the relatively low optical refractive index of *nc*-ITO (~1.8), the overall absorption is further increased to 98.5%-99% after coating the surface with *nc*-ITO (**Figure 3b**). Moreover, the excellent long wavelength transmission properties of this *nc*-ITO allow nearly-all photons to be absorbed by the Si substrate. Accordingly, nearly-all light is absorbed within the Si (due to the combination of anti-reflection properties of the contact coating layer and the optical impedance matching provided by the tapered low-aspect-ratio nanostructures) and thus leads to a corresponding generation of photocarriers.

Secondly, the *nc*-ITO forms a Schottky contact with the *n*-Si substrate, inducing energy band bending at the surface and corresponding built-in electric field. This built-in electric field (pointing towards the top surface) ensures that the photogenerated minority carriers (holes) can undergo drift transport to the *nc*-ITO contact (**Figure 1a**, **d** and **e**). On the other hand, according to Poisson's equation, the electric field strength is an integration of the fixed charges over the whole field region and this electric field strength peaks in the near-surface region (simulated in **Figure 5d**). The value of the surface electric field is dependent on both the Schottky barrier height and the doping level of the Si substrate. As a result, the *nc*-ITO contact provides considerably high surface electric field strengths to extract photocarriers and thus mitigate the photocarrier recombination loss. In addition to the induced surface electric field, the simple air-exposure and air-annealing process also help the growth of native SiO_x at the interface, providing considerably low surface trap state density and hence also mitigating the recombination rate. The contact-induced surface electric field and the chemical passivation effect of the SiO_x together contribute to the high extraction rate of these photocarriers approaching the surface. This simple strategy makes it possible for nearly-all photocarriers (approaching the surface) to be extracted as a photocurrent with minimal recombination loss and thus leading to the realization of near-100% EQE.

Thirdly, the deep-well depletion region with ~40 μm width enables most of the photocarriers corresponding to broadband light of 500 nm to 960 nm wavelength to be generated within the built-in electric field region and thus be effectively transported to the external contact

11. Surface recombination analysis

We provide a comparison of the performance of the devices fabricated with 10^{12} cm^{-3} and with 10^{15} cm^{-3} doping concentrations in **Figure S7**. The devices prepared on 10^{15} cm^{-3} doped Si clearly exhibit a higher EQE at short wavelengths. For example, for 20 nm ITO devices (same parasitic absorption within ITO), the device made on 10^{12} cm^{-3} doping level has a 75% EQE at 400 nm wavelength (black curve), while the device made on 10^{15} cm^{-3} doping level has an 85% EQE at the same wavelength (magenta curve). This clearly shows that in addition to ITO absorption, there is still some recombination loss for the devices prepared on 10^{12} cm^{-3} doping. According to Poisson's equation, the surface electric field for devices on 10^{15} cm^{-3} doping level is larger than that for devices of 10^{12} cm^{-3} doping level, explaining the lower recombination rate due to the strong surface electric field effect.

12. The exponential scale of the photocurrent

The photocurrent on an exponential scale is shown in **Figure S10**.

13. Optoelectronic characterization

Additional photosensitivity results are displayed in **Fig. S11**. The measurement is shown in **Fig. S11a**, where a green laser illuminates the device region. The linearity of photocurrent-voltage curves under various illumination powers (~10 μW to ~90 μW with 10 μW steps) at 515 nm and 980 nm is shown in **Figs. S11b** and **c**, respectively. Under small optical injection, the photocurrents saturate at 0 V bias for all two wavelengths, consistent with the EQE results in **Fig. 3d** in the manuscript. For instance, at injection powers below ~60 μW at 515 nm wavelength (**Fig. S11b**), the photocurrents are fairly consistent as the reverse voltage is increased from 0 V to -3 V; Similarly, at 980 nm (**Fig. S11c**) all photocurrents saturate at 0 V bias for injection powers below ~40 μW. These results demonstrate the viability of nano-stalagmite b-Si Schottky photodiodes to operate in a self-powered mode for the detection of weak illumination signals. Under higher optical injection, a larger reverse bias is required for the photocurrent to reach saturation level for all two wavelengths. Under -3 V bias, linearity in photocurrent with increasing power is observed for all three wavelengths (515, 780 and 980 nm wavelength, **Fig. S11d**).

14. Specific detectivity comparison with the state-of-the-art reports

The detectivity of our work is compared with the state-of-the-art high detectivity works in **Figure S12**.[10, 19-22]

15. The temporal response over a long period of time

The temporal response results under the illumination of 515 nm and 1060 nm wavelengths are shown in **Figure S13a**, and **S13b**, respectively. The results are measured over a long duration of 100 ms, under 0 V bias, showing excellent stability of broadband detection over a long period of time.

16. Discussion of potential shortcomings and possible strategies to further improve the device performance of the present work

Potential shortcomings of the present device design are discussed below. The integration of the *nc*-ITO contact enables excellent light transmission at long wavelengths from 500 nm to 960 nm, thus realizing a high EQE of up to >98% at these wavelengths. However, the parasitic absorption of *nc*-ITO unavoidably limits the performance of the photodiode at short wavelengths (observe the EQE dropping below 500 nm wavelength in **Figure 3d**), compared with the state-of-the-art *b*-Si PIN photodiodes for which the EQE is demonstrated to even exceed unity at short wavelengths.[7,23] Putting the junction at the rear side of the device may be helpful to overcome this – a topic for follow-on research.[24] Meanwhile, a Schottky junction has its natural limitation of higher reverse dark current density compared with the PIN photodiode. This severely influences further improvement of the device limit of detection of faint light or even a single photon. For example, the advanced PIN commercial photodiodes already achieve reverse dark current down to pA level with noise equivalent power (NEP) down to 10^{-15} W/ √ HZ (for example, PD1sM, PD5sMG from ElFys). We believe that a higher work function interfacial buffer layer can be inserted between *nc*-ITO and *n*-Si for follow-on research to further reduce the dark current of the device. Further, to maintain process simplicity, the rear side of the present device is unoptimized in relation to a bare Al electrode as the ohmic contact. It is suggested that this direct contact

normally causes a large contact resistance, limiting high power light detection (see the nonlinear region of the photocurrent at 980 nm wavelength in **Figure 4d**). In the absence of a heavily doped *n*-type rear side (for example, by high-temperature diffusion process), a low work function tunnelling contact can be explored as the buffer layer between *n*-Si and Al to reduce the contact resistance – a study for the future.

17. Schottky barrier measurement and energy band diagram, and carrier concentration simulation

Capacitance-voltage (C-V) curves obtained at various frequencies (**Fig. S14a**) and the $1/C^2$-V curves at 5 kHz and 10 kHz (**Fig. S14b**) show a horizontal axis intercept of ~0.25 V, indicating an effective barrier height of ~0.71 eV from ITO to b-Si. The modelled energy band diagram at an applied bias of -3 V is plotted as a function of device thickness (~400 µm) in **Fig. S14c**, under no illumination. A depletion region width of ~90 µm is obtained at -3 V bias. The quasi-Fermi levels for both electron and hole under -3 V bias are given in **Fig. S14c**. The carrier concentration across the entire wafer under different biases is shown in **Fig. S14d**. We observe a slight inversion region at the surface with hole and electron concentrations of ~10^{12} cm^{-3} and ~10^{7} cm^{-3}, respectively. With increasing bias, the depletion width increases to ~40 µm, and the free carrier concentrations are as low as ~10^{5} cm^{-3} (holes) and ~10^{7} cm^{-3} (electrons). The low concentration of both carrier types provides for a lower bulk Auger recombination rate, partially contributing to the nearly complete collection of all photocarriers at 0 V bias under low optical injection.

18. Heart rate monitor

The heart rate monitor set up is shown in **Fig. S15**, which is discussed in the experimental section in the manuscript. With the green light, the heart rate monitor exhibits sensitivity with a mere 1 µW of illumination power (**Fig. S16a**) or a faint source emitting only 200 nW power (**Fig. S16b**). For 970 nm LED, the heart rate sensor shows the accurate measurement for illumination power levels at 500 nW (**Fig. S16c**). The enhanced photocarrier transport and the associated high detectivity at long wavelengths (approaching 10^{14} Jones at 970 nm, **Fig. 4e** in the manuscript) enable superior heart-rate sensing under faint illumination, even at long wavelengths where the

human blood is weak absorption.

The LED wavelength and the LED power used for the heart rate sensor in this work are compared with those of other reported heart rate sensor studies, as well as with a commercial heart rate sensor in **Table S2**.[25-28] There are two advantages of the heart rate sensor in this work compared with other sensors. First, the heart rate sensor can respond to broadband of wavelengths (LEDs of 470 nm to 1050 nm wavelength). This is attributed to the high broadband QE of the *b*-Si photodiode. As highlighted in the manuscript, a broadband wavelength-based heart rate sensor is helpful in achieving more accurate measurements since the range of blood components have a range of resonant frequency. Secondly, the high QE of the *b*-Si photodiode also makes it possible for low-power consuming LED to be used within the heart rate sensor. This is another significant advantage of the heart rate sensor demonstrated herein compared to other sensors. For a portable heart rate measurement system, one of the major power-consuming components is the LED light source. For example, a commercial heart rate sensor is usually programmed to record the individual's heart rate and concurrently the operation of the LED light source. At present, commercial heart rate sensors usually do not exhibit long operating times – typically requiring recharging of the limited capacity battery in relation to the power draw of the sensor. The demonstrated high QE of the present photodiode implied that the device effectively converts all light essentially into an electrical signal, and as such, is expected to markedly lower the demand for electrical power and thus enable a long operating time for a portable heart rate sensor.

19. Signal to noise ratio for the calibrated photodetectors

The signal-to-noise ratio for the calibrated reference photodetectors before EQE measurements is given in **Figure S17**.

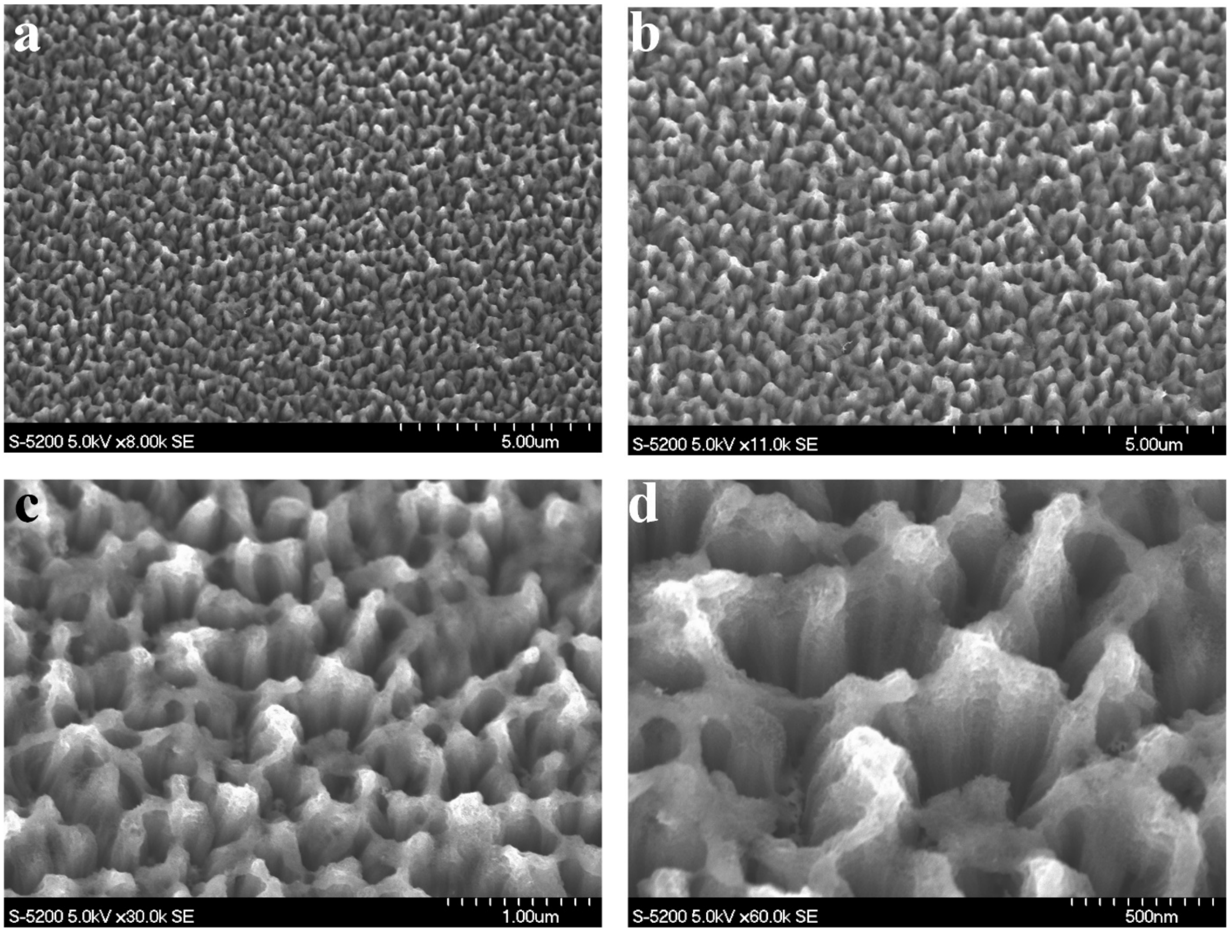

Figure S1. SEM images (top view) of the nanostructures prior to AuNP removal. Magnification level **(a)** ×8 K, **(b)** ×11 K **(c)** ×30 K and **(d)** ×60 K.

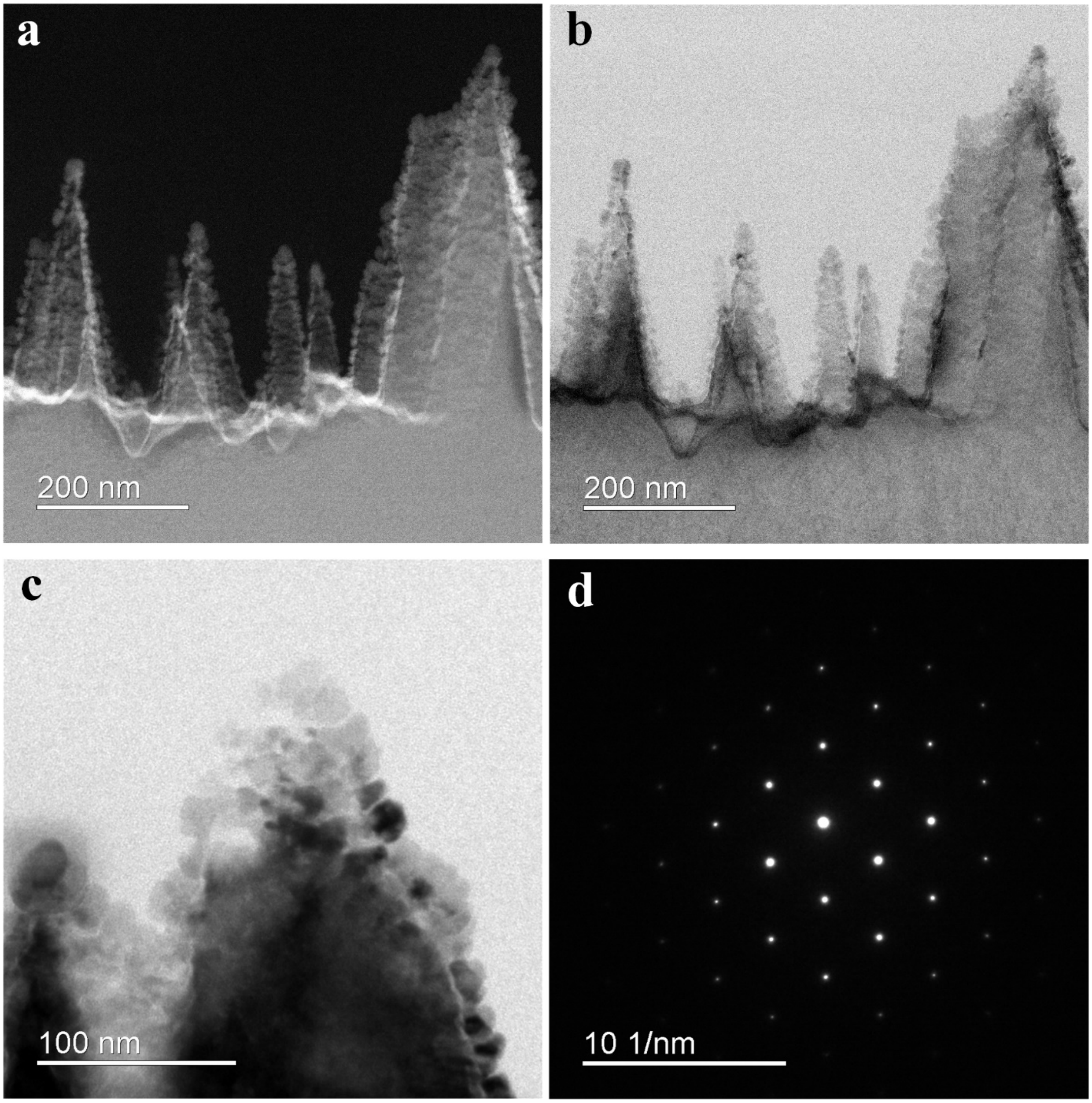

Figure S2. HRTEM images for 20 nm ITO onto nano-stalagmites. **(a)** Light field TEM image for an array of nano-stalagmites **(b)** Dark field TEM image for an array of nano-stalagmites. **(c)** TEM image for a sole nc-ITO/nano-stalagmite, corresponding to the diffraction point image in **Figure 2(j)** in the manuscript. **(d)** Diffraction point image for single-crystalline Si.

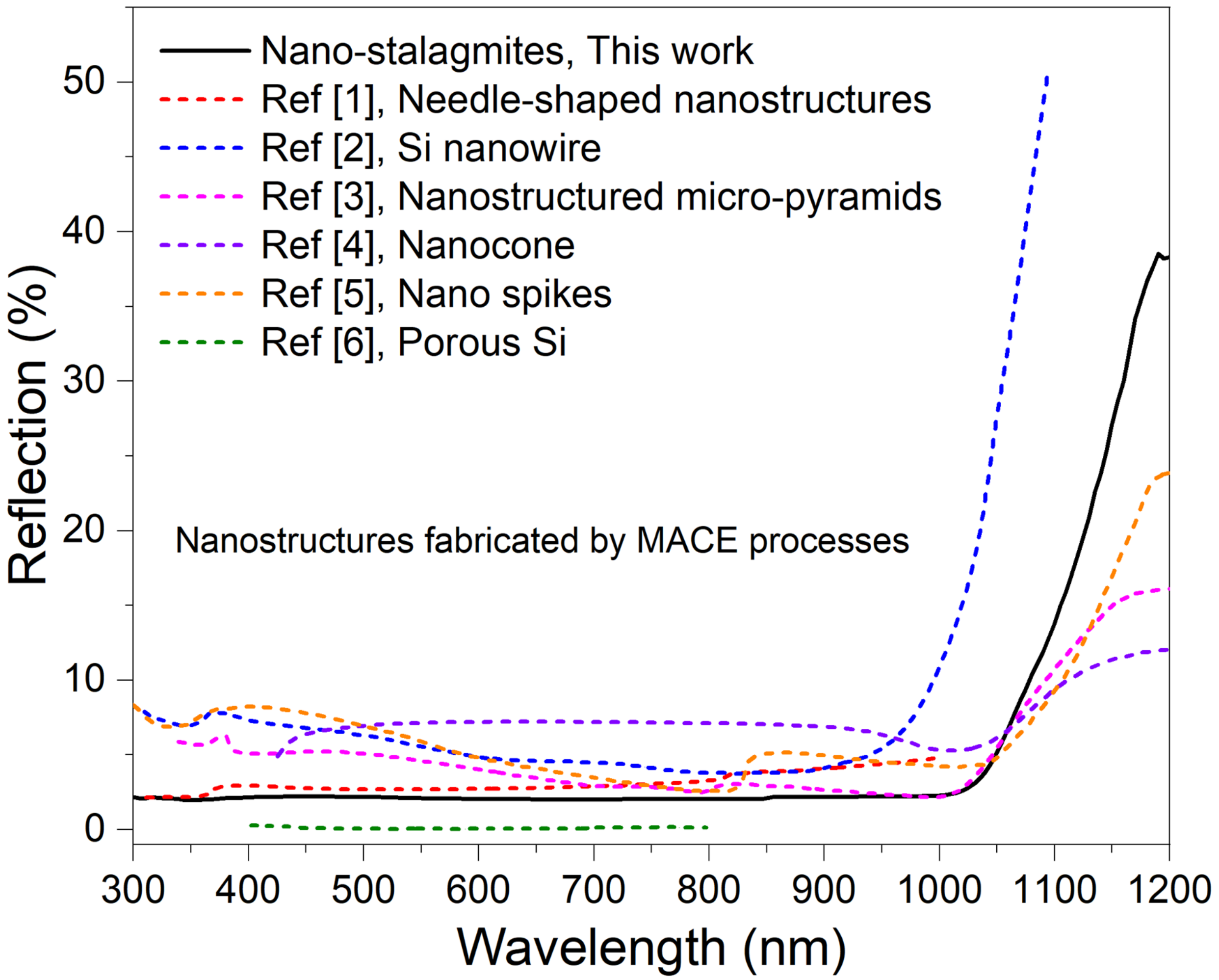

Figure S3. Optical reflection of nano-stalagmite *b*-Si in this work, in comparison with some of the reported nanostructures fabricated by metal-assisted chemical etching (MACE) processes.

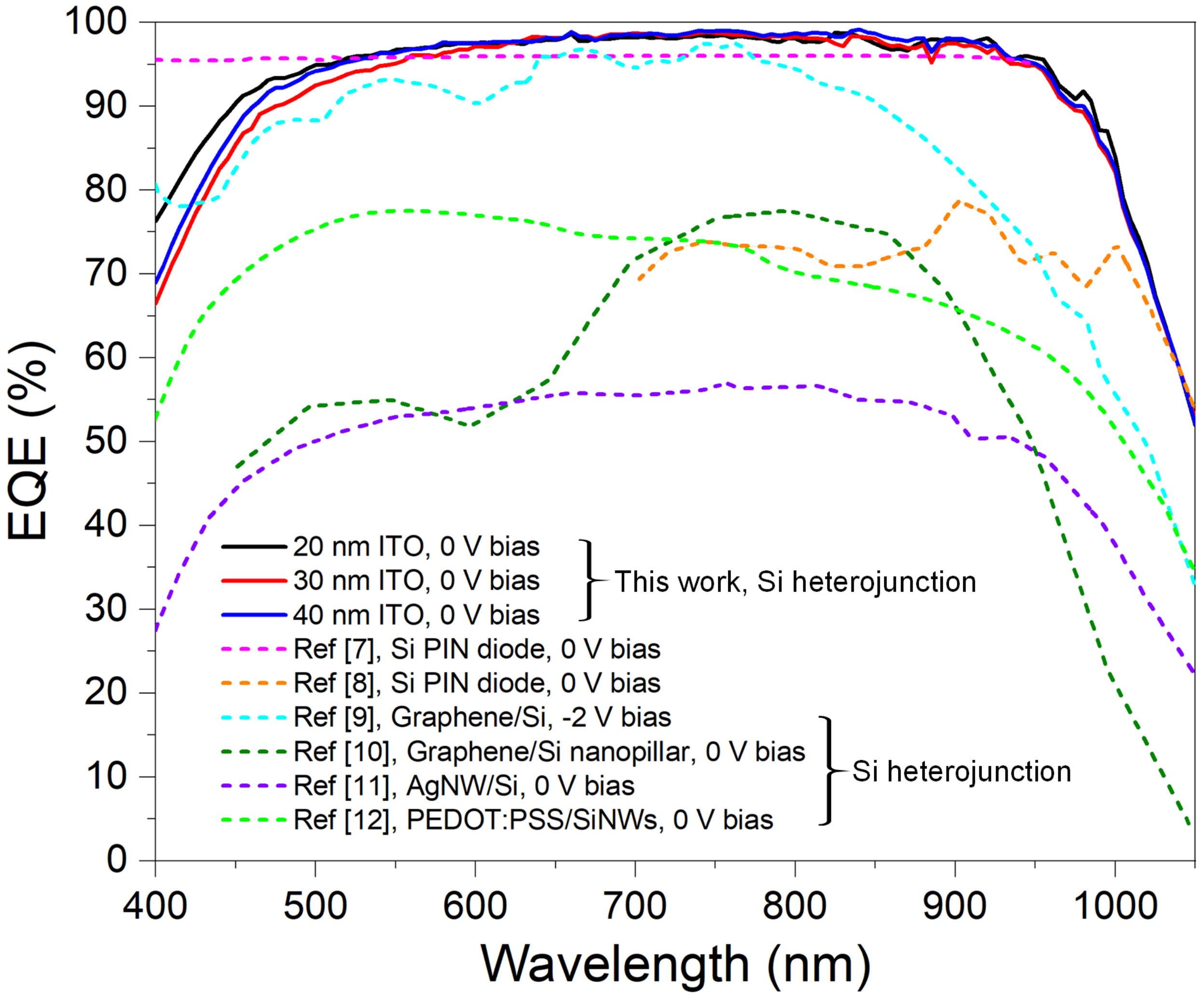

Figure S4. External quantum efficiency (EQE) of nano-stalagmite *b*-Si photodiode having different ITO thicknesses compared with some of the reported state-of-the-art general photodiodes and Si heterojunction photodiodes.

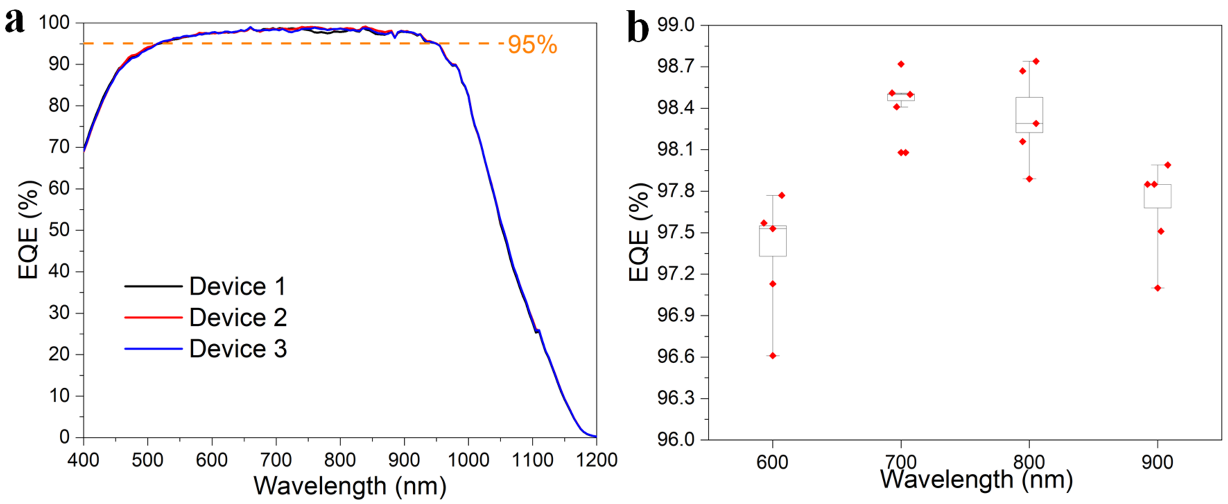

Figure S5. Repeatability of EQE **(a)** EQE measured in 3 separate devices from 400 nm to 1200 nm wavelength. (b) EQE statistics from various devices at four wavelengths: 600 nm, 700 nm, 800 nm and 900 nm.

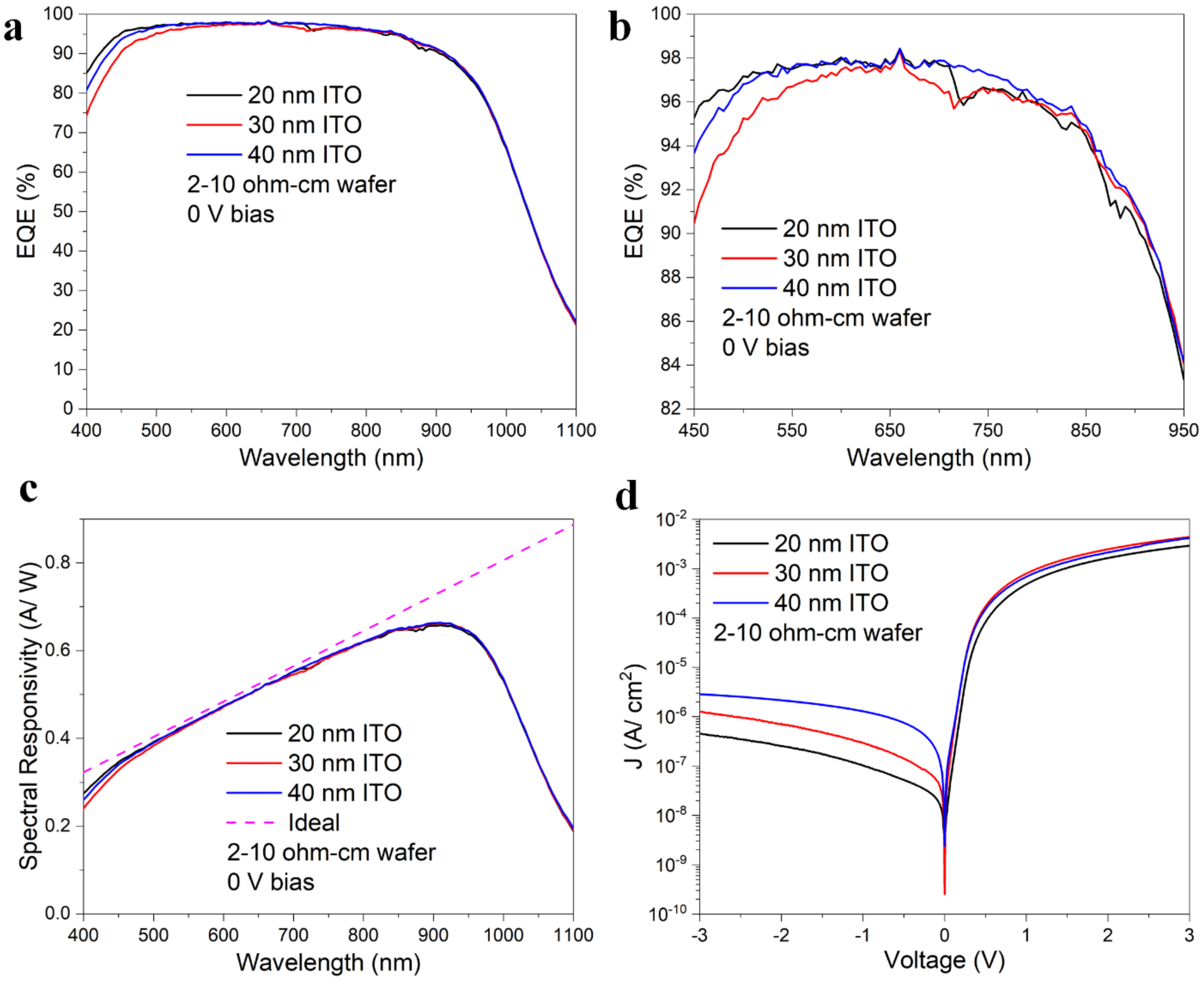

Figure S6. Device performance for 'near-field' device **(a)** EQE curves for devices made on 2-10 ohm-cm silicon with various ITO thicknesses. **(b)** Zoomed-in view of the EQE curves in (a), where the vertical scale ranges from 82% to 100%. **(c)** Spectral responsivity for devices made on 2-10 ohm-cm Si with various ITO thicknesses. **(d)** Dark current density of devices made on 2-10 ohm-cm *n*-Si.

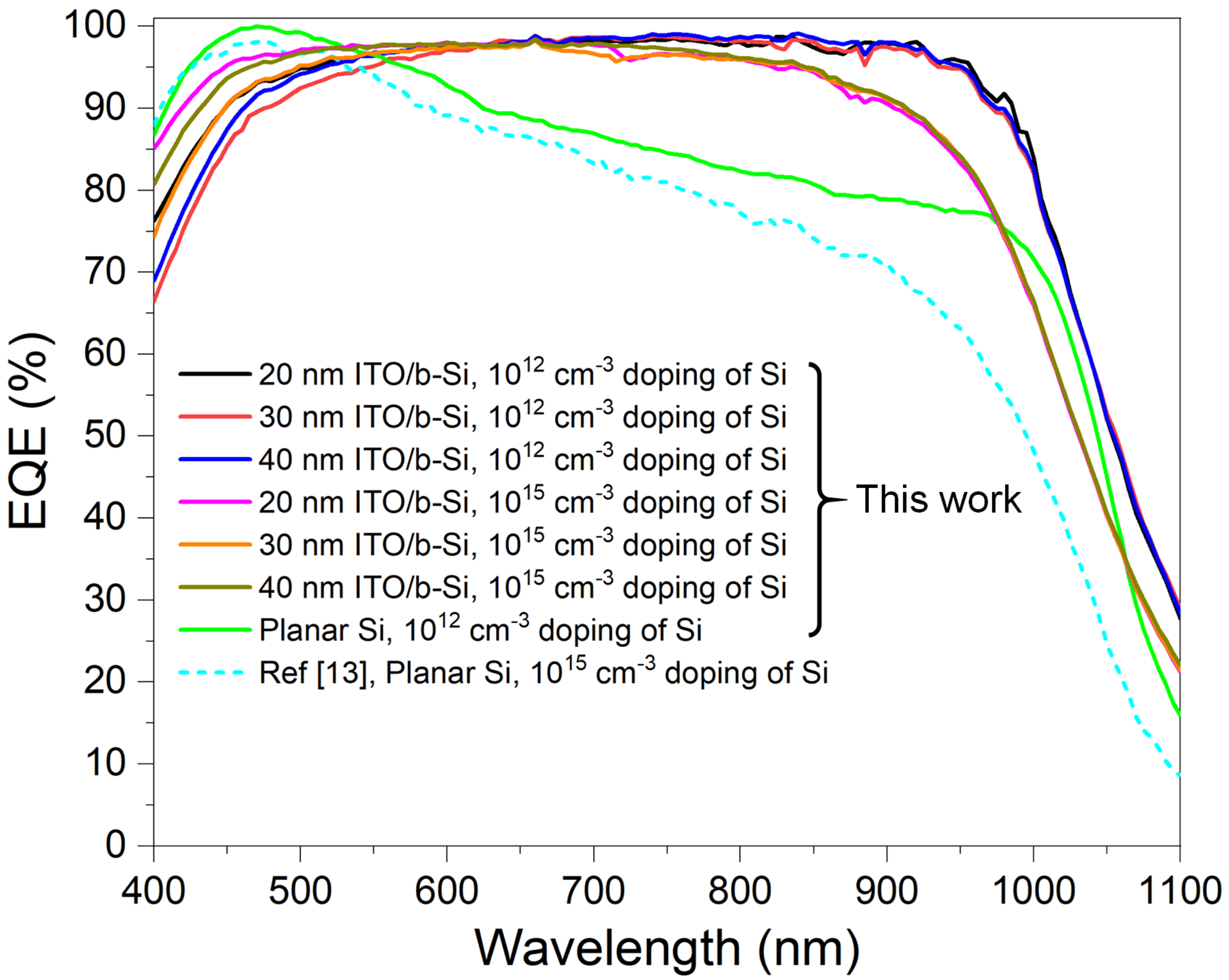

Figure S7. Comparison of EQE of nanostructured Si and planar Si devices, clearly illustrating the significant advance of the present work where the EQE is approaching the upper limit of 100% over a broadband of wavelengths.

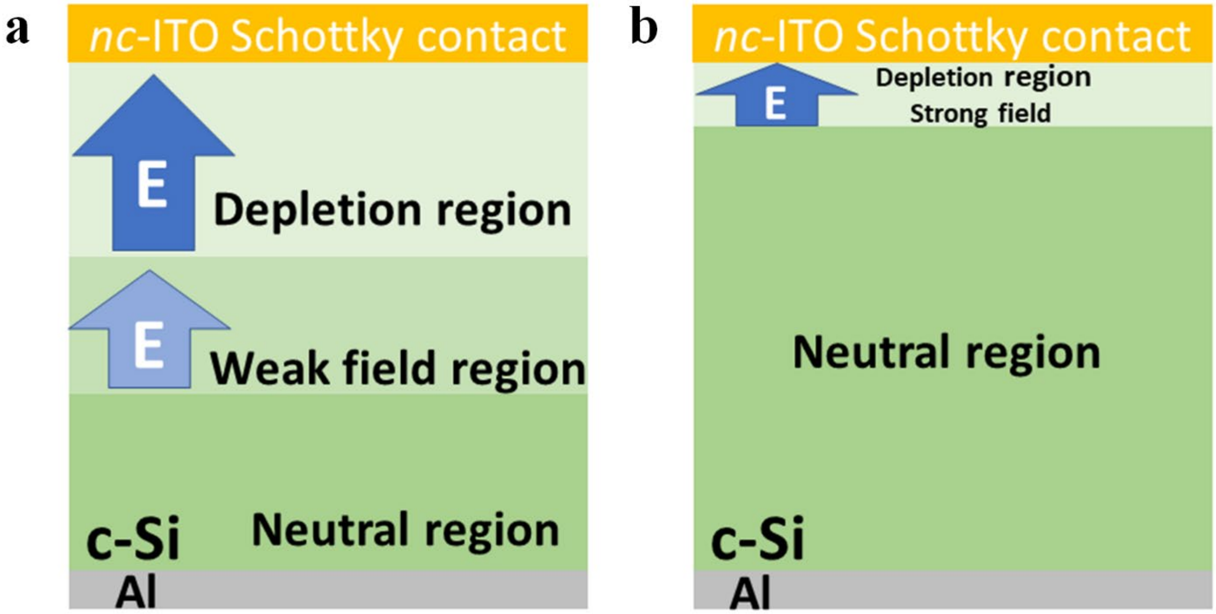

Figure S8. Comparison of the electric field regions of devices fabricated from 3000 ohm-cm and 3 ohm-cm silicon. **(a)** Schematic illustration of the field region where we show relatively high and low electric field regions, as well as the neutral region, in the 3000 ohm-cm resistivity Si based device. **(b)** Schematic illustration of the narrow depletion region with a strong electric field, as well as the large neutral region, in devices with 3 ohm-cm resistivity Si.

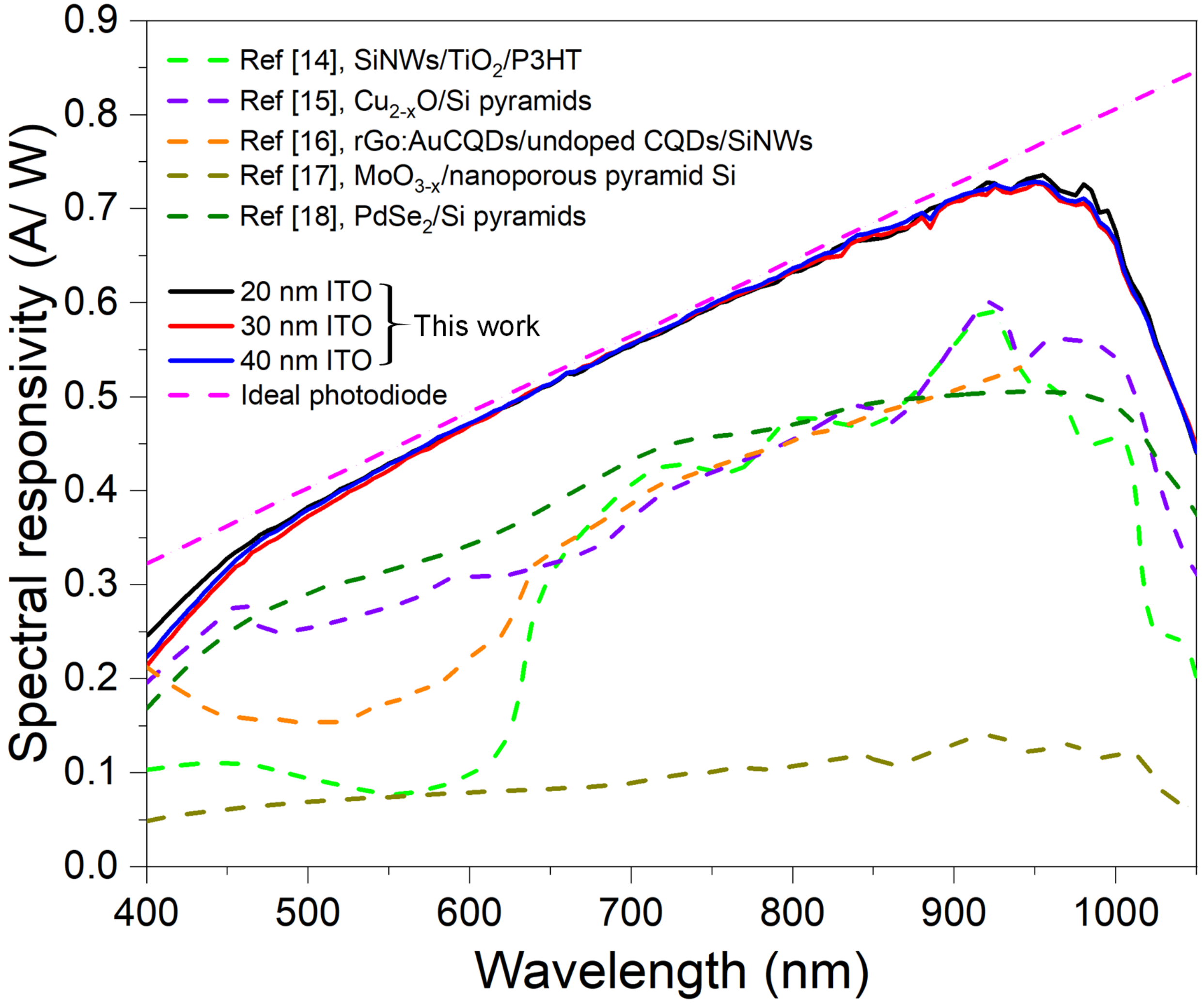

Figure S9. Spectral responsivity of nano-stalagmite *b*-Si photodiode having different ITO thicknesses, compared with some of the reported heterojunction photodiodes.

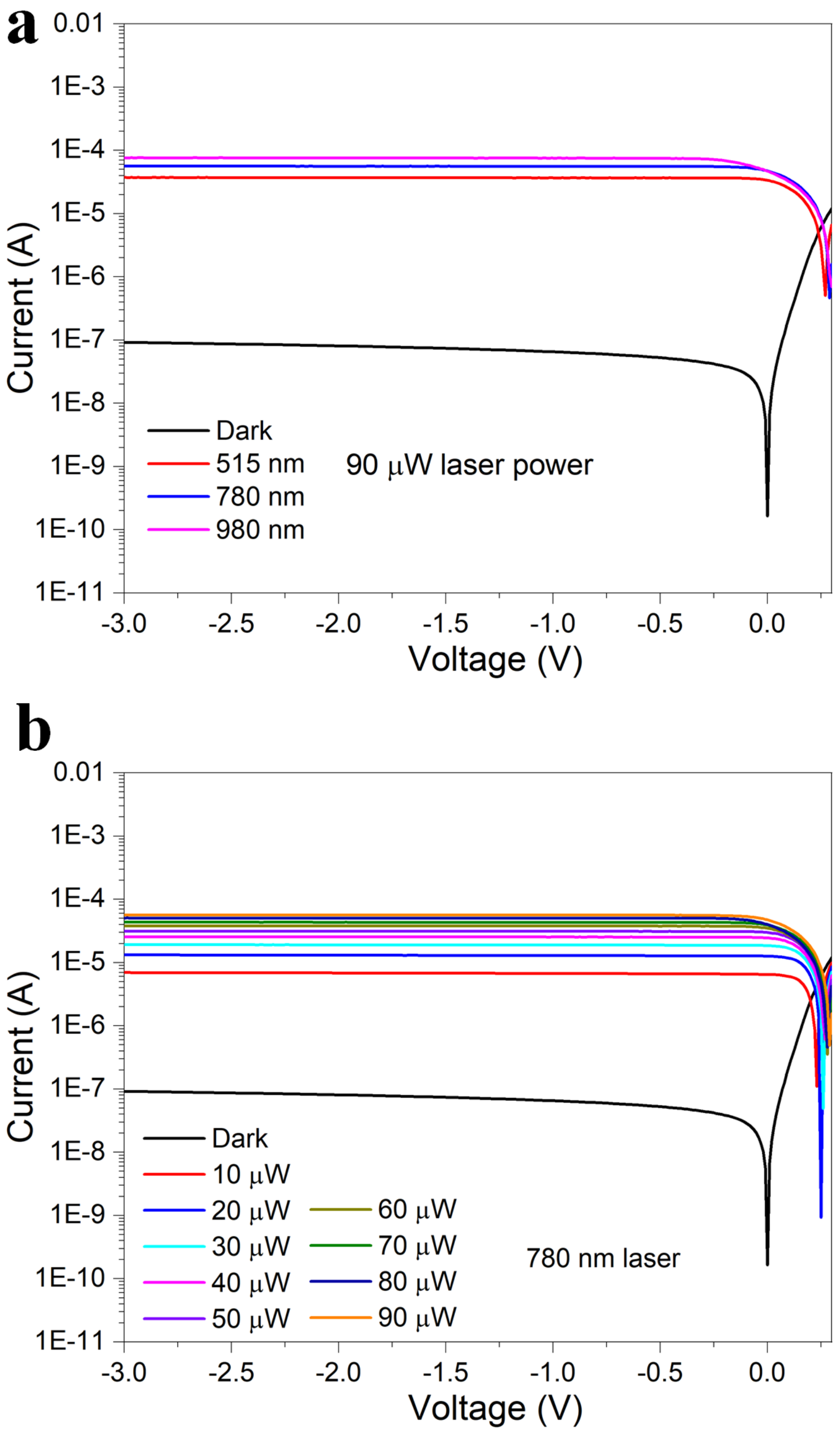

Figure S10. Log scale plot of the photocurrent. **(a)** Log scale photocurrent at different wavelengths. **(b)** Log scale photocurrents at varying laser powers for 780 nm wavelength laser.

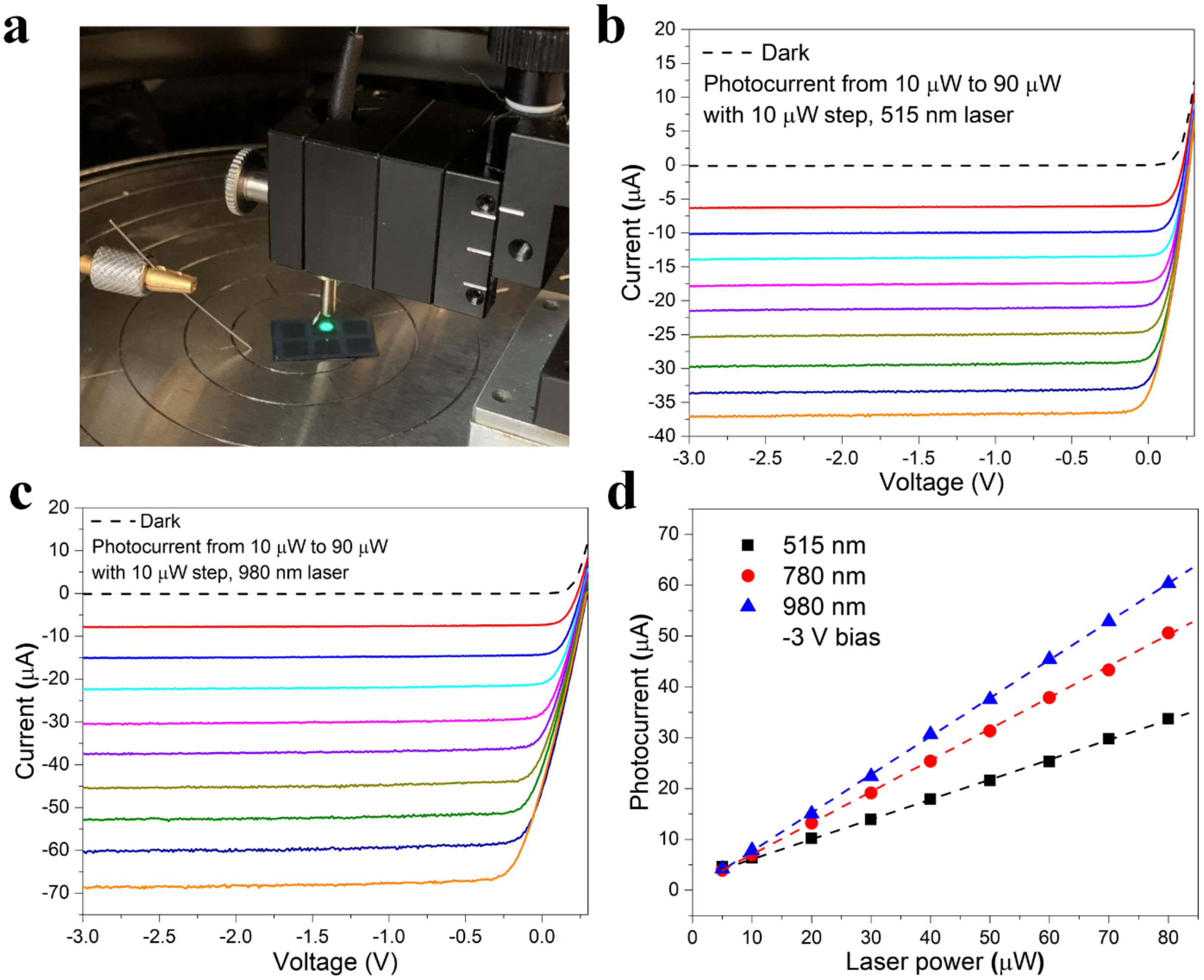

Figure S11. Additional photocurrent characterization of the devices. **(a)** Optical photograph of the photocurrent measurement apparatus. **(b)** I-V curves measured under different laser powers (from 10 μW to 90 μW with 10 μW steps) using 515 nm wavelength laser. **(c)** I-V curves measured under different laser powers (from 10 μW to 90 μW with 10 μW steps) using 980 nm wavelength laser. **(d)** Photocurrent as a function of laser power under various wavelength illumination at -3 V bias.

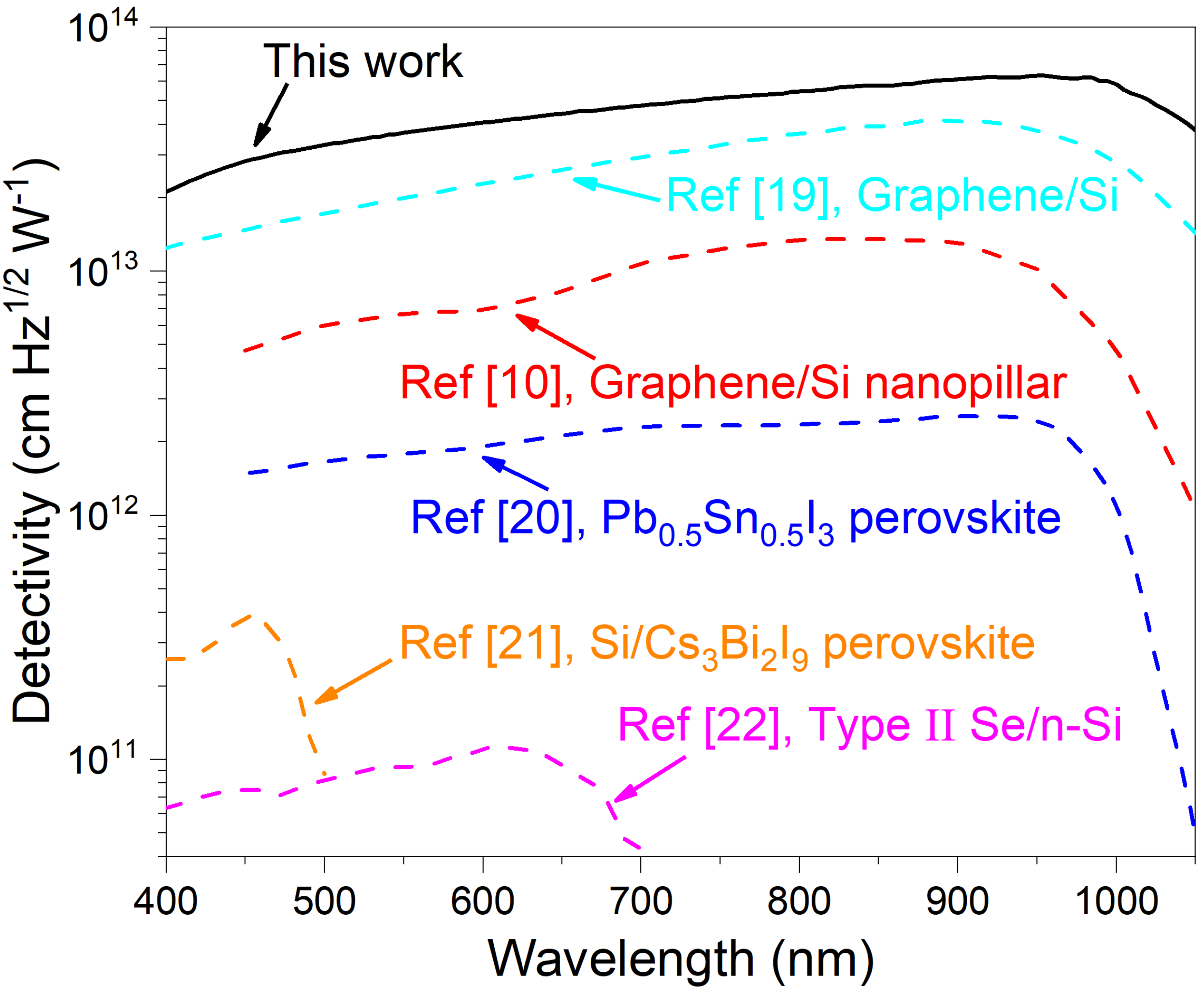

Figure S12. Specific detectivity compared with some of the reported high-detectivity heterojunction photodiodes.

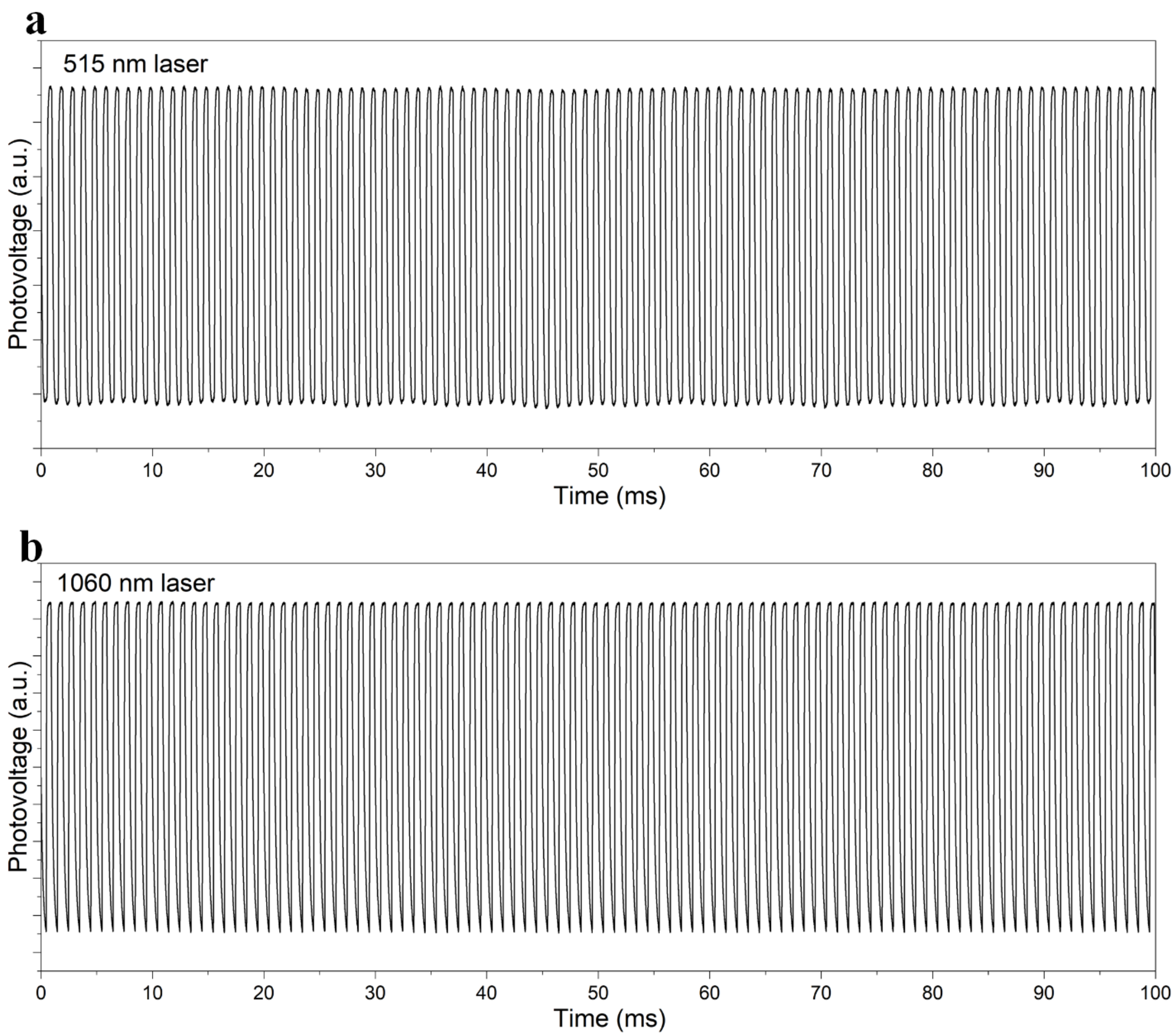

Figure S13. Temporal response over a large time scale. (a) Temporal response for 515 nm laser illumination at 0 V. (b) Temporal response for 1060 nm laser illumination at 0 V.

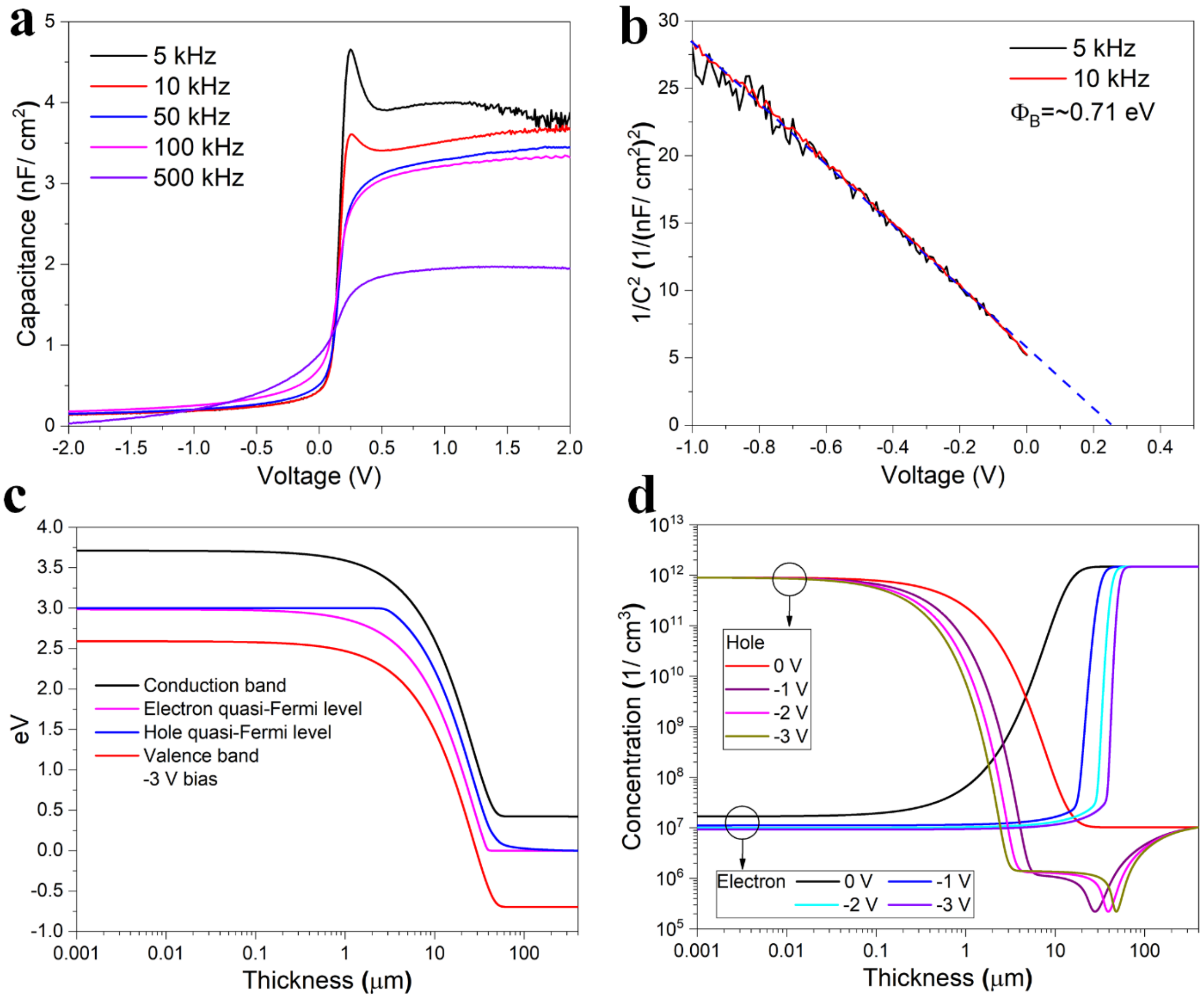

Figure S14. Schottky barrier height measurement and electrical simulations for the *b*-Si device. **(a)** Capacitance-voltage (C-V) curves measured under various frequencies ranging from 5 kHz to 500 kHz. **(b)** $1/C^2$-V fitting curve. **(c)** Simulated device energy band diagram at -3 V bias. **(d)** Simulated carrier concentration under different reverse biases across the thickness of the wafer.

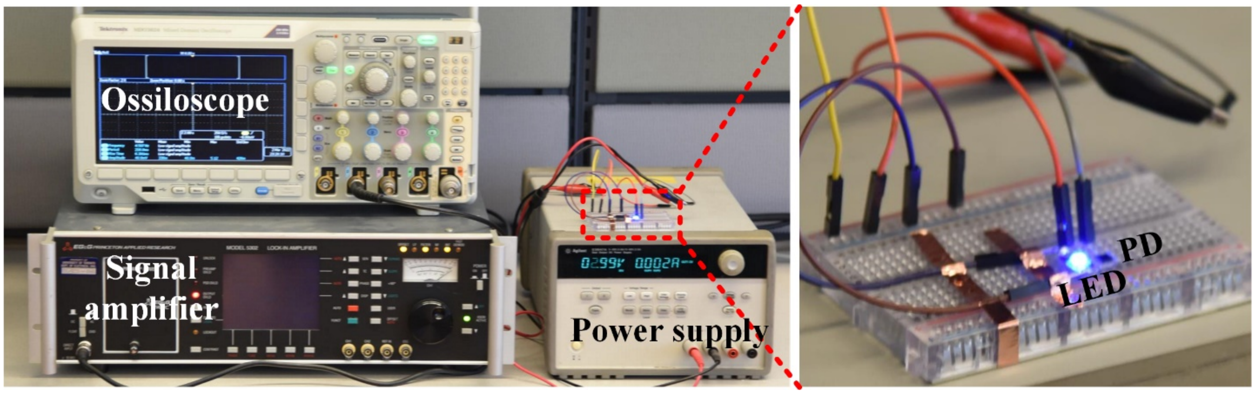

Figure S15. Photograph of the test apparatus for the heart rate sensor.

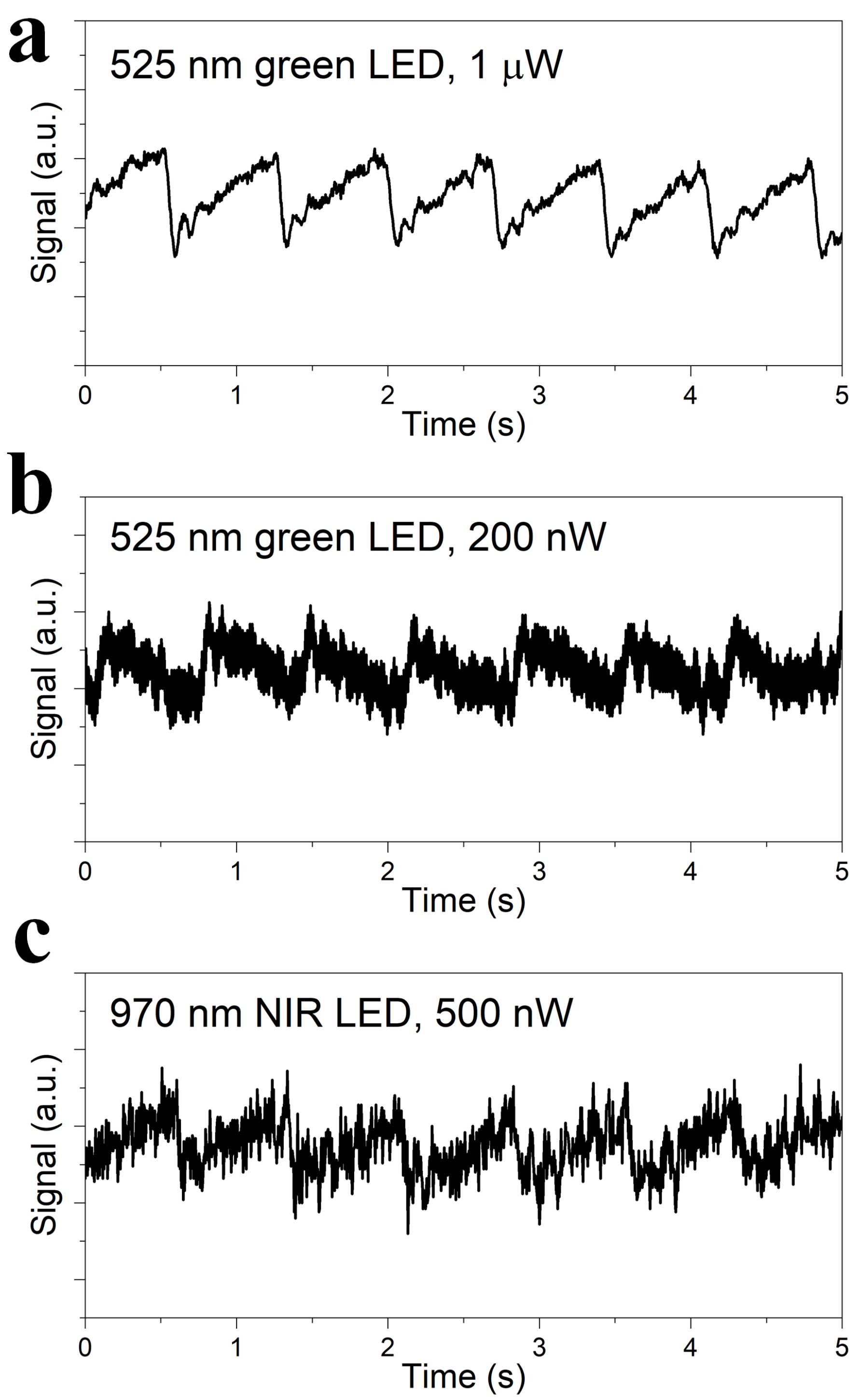

Figure S16. Additional heart rate test results. **(a)** Heart rate signal under weak illumination (green LED, 1 μW). **(b)** Heart rate signal under faint illumination (green LED, 200 nW). **(c)** Heart rate signal under weak NIR illumination (970 nm, 500 nW).

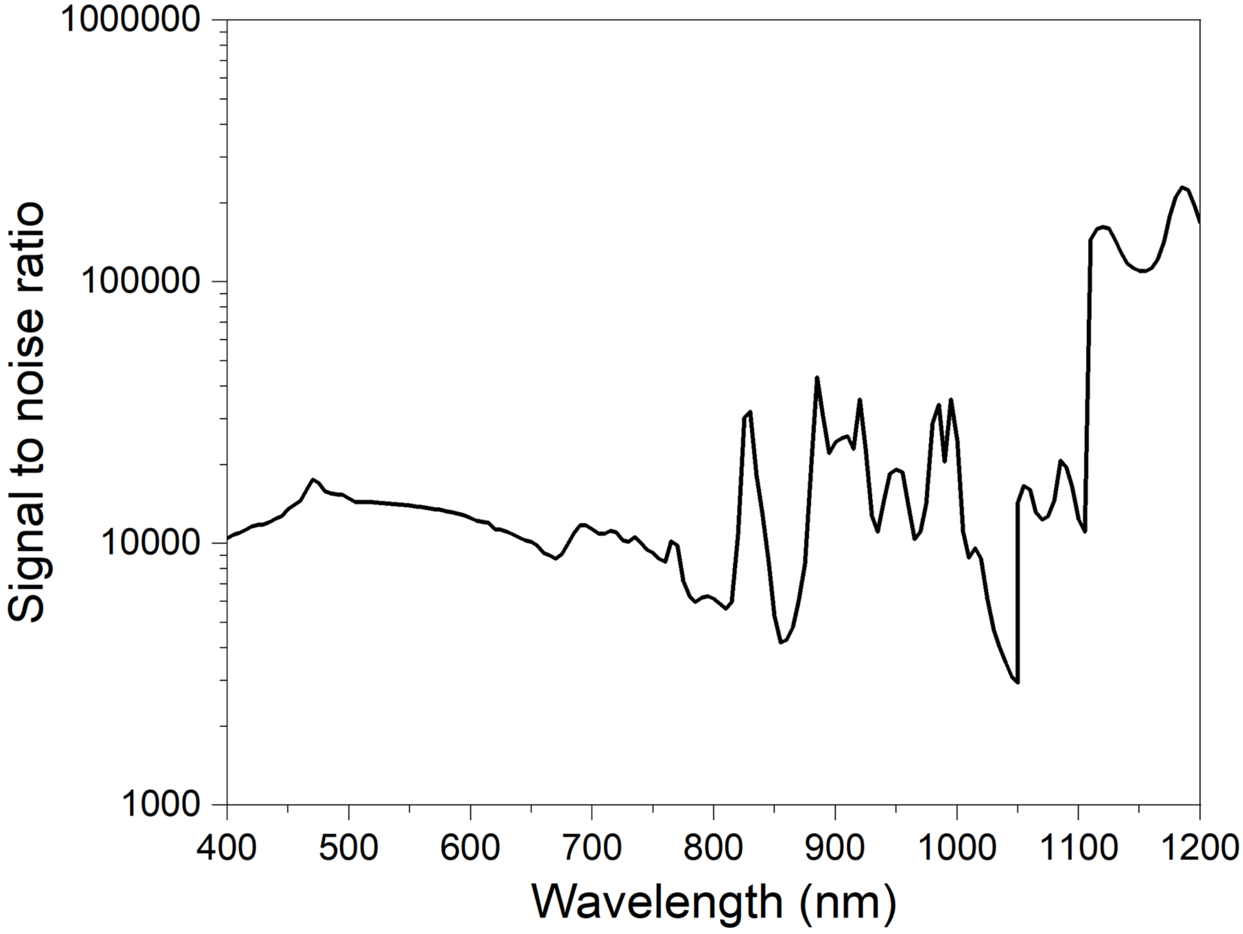

Figure S17. Signal to noise ratio for the calibrated photodetectors.

Table S1. Detailed comparison between the photodiodes in this work and the state-of-the-art devices vis-à-vis light trapping/anti-reflection structures, dark current density and the fabrication technology.

Diode type	Nano-structures	Dark current density (A/ cm^2)	Fabrication technology	Reference
Single *nc*-ITO/ *b*-Si Schottky photodiode	Nano stalagmites	1.3×10^{-7} at -1 V	Wet etching, PVD, 400 °C air-annealing.	This work
PIN diode with induced junction	Nano needles	1.66×10^{-8} at -1 V	RIE, ion implication, high temperature annealing, ALD, PVD	Ref. 7
PIN diode	Hourglass shaped nanowire	The order of 10^{-6} at -0.5 V	Ion implication, high temperature annealing, photolithography, ICP-RIE, CVD, PVD	Ref. 8
Graphene/Si Schottky photodiode	N/A	The order of 10^{-9} A at -2 V, (effective area N/A)	High temperature annealing, photolithography, PVD, CVD, graphene transfer	Ref. 9
Graphene/Si Schottky photodiode	nanopillars	The order of 10^{-8} A at -0.4 V (effective area N/A)	Ion implantation, E-beam lithography, dry etching, PVD, graphene transfer, 500 °C annealing	Ref. 10
AgNW/p-Si Schottky photodiode	N/A	10^{-3} at -1.5 V bias	Solution process of AgNW, PVD	Ref. 11

Table S2. Detailed comparison between the heart rate sensor in this work and reported heart rate systems/sensors vis-à-vis LED wavelength and LED power.

Photodiode type	LED wavelengths	LED power	Reference
b-Si heterojunction PD	470nm, 525nm, 625nm, 970 nm, 1060 nm	From 250 µW down to 100 nW	This work
Organic photodiode (OPD)	635 nm	26 µW	Ref. 25
OPD	680 nm	8 mW	Ref. 26
OPD	630 nm, 860 nm	35 µW	Ref. 27
Si PD	Green and red LEDs	2 µW	Ref. 28
MAX30101	880 nm, 660 nm, 537 nm	6.5 mW to 17.2 mW	Commercial system

References

[1] K. Chen, T. P. Pasanen, V. Vähänissi, H. Savin, *IEEE J. Photovoltaics* **2019**, *9*, 974.

[2] H. D. Omar, M. R. Hashim, M. Z. Pakhuruddin, *Opt. Laser Technol.* **2021**, *136*, 106765.

[3] F. Toor, J. Oh, H. M. Branz, *Prog. Photovoltaics Res. Appl.* **2015**, *23*, 1375.

[4] H. Zhong, A. Guo, G. Guo, W. Li, Y. Jiang, *Nanoscale Res. Lett.* **2016**, *11*, 322.

[5] T. Zhang, P. Zhang, S. Li, W. Li, Z. Wu, Y. Jiang, *Nanoscale Res. Lett.* **2013**, *8*, 351.

[6] C. F. Guo, T. Sun, Y. Wang, J. Gao, Q. Liu, K. Kempa, Z. Ren, *Small* **2013**, *9*, 2415.

[7] M. A. Juntunen, J. Heinonen, V. Vähänissi, P. Repo, D. Valluru, H. Savin, *Nat. Photonics* **2016**, *10*, 777.

[8] K. Kim, S. Yoon, M. Seo, S. Lee, H. Cho, M. Meyyappan, C. K. Baek, *Nat. Electron.* **2019**, *2*, 572.

[9] S. Riazimehr, S. Kataria, J. M. Gonzalez-Medina, S. Wagner, M. Shaygan, S. Suckow, F. G. Ruiz, O. Engström, A. Godoy, M. C. Lemme, *ACS Photonics* **2019**, *6*, 107.

[10] B. Feng, X. Pan, T. Liu, S. Tian, T. Wang, Y. Chen, *Nano Lett.* **2021**, *21*, 5655.

[11] M. Kumar, M. Patel, H. S. Kim, J. Kim, J. Yi, *ACS Appl. Mater. Interfaces* **2017**, *9*, 38824.

[12] Z. Liang, P. Zeng, P. Liu, C. Zhao, W. Xie, W. Mai, *ACS Appl. Mater. Interfaces* **2016**, *8*, 19158.

[13] Y. Zhang, J. Y. Y. Loh, A. G. Flood, C. Mao, G. Sharma, N. P. Kherani, *Adv. Funct. Mater.* **2022**, *32*, 2109794.

[14] L. Chen, W. Tian, C. Sun, F. Cao, L. Li, *ACS Appl. Mater. Interfaces* **2019**, *11*, 3241.

[15] Y. Liu, J. Zhu, G. Cen, J. Zheng, D. Xie, Z. Zhao, C. Zhao, W. Mai, *ACS Appl. Mater. Interfaces* **2019**, *11*, 43376.

[16] K. Sarkar, P. Devi, A. Lata, V. K. Lokku, P. Kumar, *Adv. Opt. Mater.* **2020**, *8*, 2000228.

[17] Y. Liu, G. Cen, G. Wang, J. Huang, S. Zhou, J. Zheng, Y. Fu, C. Zhao, W. Mai, *J. Mater.*

Chem. C **2019**, *7*, 917.

[18] F. X. Liang, X. Y. Zhao, J. J. Jiang, J. G. Hu, W. Q. Xie, J. Lv, Z. X. Zhang, D. Wu, L. B. Luo, *Small* **2019**, *15*, 1903831.

[19] X. Li, M. Zhu, M. Du, Z. Lv, L. Zhang, Y. Li, Y. Yang, T. Yang, X. Li, K. Wang, H. Zhu, Y. Fang, *Small* **2016**, *12*, 595.

[20] R. Ollearo, J. Wang, M. J. Dyson, C. H. L. Weijtens, M. Fattori, B. T. van Gorkom, A. J. J. M. van Breemen, S. C. J. Meskers, R. A. J. Janssen, G. H. Gelinck, *Nat. Commun.* **2021**, *12*, 7277.

[21] Z. Li, X. Liu, C. Zuo, W. Yang, X. Fang, *Adv. Mater.* **2021**, *33*, 2103010.

[22] W. Yang, K. Hu, F. Teng, J. Weng, Y. Zhang, X. Fang, *Nano Lett.* **2018**, *18*, 4697.

[23] M. Garin, J. Heinonen, L. Werner, T. P. Pasanen, V. Vähänissi, A. Haarahiltunen, M. A. Juntunen, H. Savin, *Phys. Rev. Lett.* **2020**, *125*, 117702.

[24] A. Richter, R. Müller, J. Benick, F. Feldmann, B. Steinhauser, C. Reichel, A. Fell, M. Bivour, M. Hermle, S. W. Glunz, *Nat. Energy* **2021**, *6*, 429.

[25] C. Fuentes-Hernandez, W. F. Chou, T. M. Khan, L. Diniz, J. Lukens, F. A. Larrain, V. A. Rodriguez-Toro, B. Kippelen, *Science* **2020**, *370*, 698.

[26] Y. Xia, L. E. Aguirre, X. Xu, O. Inganäs, *Adv. Electron. Mater.* **2020**, *6*, 1901017.

[27] H. Lee, W. Lee, H. Lee, S. Kim, M. V. Alban, J. Song, T. Kim, S. Lee, S. Yoo, ACS Photonics 2021, 8, 3564.

[28] A. Caizzone, A. Boukhayma, C. Enz, *IEEE Trans. Biomed. Circuits Syst.* **2019**, *13*, 1243.
